# Ibuprofen and Diclofenac Restrict Migration and Proliferation of Human Glioma Cells by Distinct Molecular Mechanisms

**DOI:** 10.1371/journal.pone.0140613

**Published:** 2015-10-20

**Authors:** Verena Leidgens, Corinna Seliger, Birgit Jachnik, Tobias Welz, Petra Leukel, Arabel Vollmann-Zwerenz, Ulrich Bogdahn, Marina Kreutz, Oliver M. Grauer, Peter Hau

**Affiliations:** 1 Department of Neurology and Wilhelm Sander-NeuroOncology Unit, University Hospital Regensburg, Regensburg, Germany; 2 Department of Neurology, Molecular Cell Biology Laboratory, University Hospital Regensburg, Regensburg, Germany; 3 Department of Internal Medicine III, University Hospital Regensburg, Regensburg, Germany and Regensburg Center for Interventional Immunology (RCI), Regensburg, Germany; University of Pécs Medical School, HUNGARY

## Abstract

**Background:**

Non-steroidal anti-inflammatory drugs (NSAIDs) have been associated with anti-tumorigenic effects in different tumor entities. For glioma, research has generally focused on diclofenac; however data on other NSAIDs, such as ibuprofen, is limited. Therefore, we performed a comprehensive investigation of the cellular, molecular, and metabolic effects of ibuprofen and diclofenac on human glioblastoma cells.

**Methods:**

Glioma cell lines were treated with ibuprofen or diclofenac to investigate functional effects on proliferation and cell motility. Cell cycle, extracellular lactate levels, lactate dehydrogenase-A (LDH-A) expression and activity, as well as inhibition of the Signal Transducer and Activator of Transcription 3 (STAT-3) signaling pathway, were determined. Specific effects of diclofenac and ibuprofen on STAT-3 were investigated by comparing their effects with those of the specific STAT-3 inhibitor STATTIC.

**Results:**

Ibuprofen treatment led to a stronger inhibition of cell growth and migration than treatment with diclofenac. Proliferation was affected by cell cycle arrest at different checkpoints by both agents. In addition, diclofenac, but not ibuprofen, decreased lactate levels in all concentrations used. Both decreased STAT-3 phosphorylation; however, diclofenac led to decreased c-myc expression and subsequent reduction in LDH-A activity, whereas treatment with ibuprofen in higher doses induced c-myc expression and less LDH-A alteration.

**Conclusions:**

This study indicates that both ibuprofen and diclofenac strongly inhibit glioma cells, but the subsequent metabolic responses of both agents are distinct. We postulate that ibuprofen may inhibit tumor cells also by COX- and lactate-independent mechanisms after long-term treatment in physiological dosages, whereas diclofenac mainly acts by inhibition of STAT-3 signaling and downstream modulation of glycolysis.

## Introduction

Glioblastomas (GBM) are characterized as highly malignant brain tumors hallmarked by infiltrating tumor cells, enhanced mitotic activity, and angiogenesis. Despite combined therapy approaches, the median survival time following diagnosis is limited to 14.6 months with standard therapy [1]. Therefore, augmentative therapies are sought for these tumors.

Non-steroidal anti-inflammatory drugs (NSAIDs) such as ibuprofen and diclofenac may be candidates for such an approach. Recent data indicate that treatment with NSAIDs reduces the risk of various cancer types [2–7] and lessens tumor growth in established tumors [2,8,9]. Traditional NSAIDs are non-selective COX-1 and -2 inhibitors. Non-selective inhibition of COX-2 leads to decreased prostaglandin synthesis [10] and prostaglandin E2 was associated with tumor cell promotion [11,12]. In addition, inhibition of tumor cell proliferation [6] and induction of apoptosis by NSAID treatment [13,14] has also been described to occur via COX-independent mechanisms.

We have previously shown that diclofenac, which preferentially inhibits COX-2 [15,16], caused c-myc inhibition followed by decreased gene expression of glucose transporter 1 (GLUT-1), as well as decreased LDH-A, and lactate secretion [17]. In addition, previous work in our lab demonstrated that diclofenac inhibits STAT-3 phosphorylation and lactate formation, induces cell cycle arrest at G2/M, and delays tumor growth in an *in vivo* animal model [18]. Diclofenac also influences the mitochondrial adenine nucleotide transferase as well as the OXPHOS complex V (F_0_F_1_-ATPase). This leads to decoupling of oxidative phosphorylation, which reduces ATP generation and thus cell proliferation [16]. In neuroblastoma, diclofenac enhanced chemotherapy induced apoptosis by augmentation of p53 [19]. It has also been determined that diclofenac treatment of neuroblastoma xenografts significantly reduced tumor growth in nude rats [8].

GBM are able to metabolically switch from the oxidative to the glycolytic pathway, a characteristic of these highly proliferative tumors [20]. Glucose is transported into the cell by glucose transporters to allow cytosolic glycolysis [21]. LDH-A catalyzes the conversion of pyruvate into lactate [22], which is then exported out of the cell by monocarboxylate transporters (MCT) [23]. High glycolytic activity is accompanied by increased extracellular lactate levels that are associated with poorer patient survival in cases of malignant glioma [24]. Glycolysis can be stimulated by oncogenes such as c-myc [25], and c-myc itself is a direct target of the master regulator STAT-3 [26].

Oxidative phosphorylation may serve as an alternative energetic pathway in tumor cells, but diclofenac has also been shown to inhibit OXPHOS [16]. Diclofenac may, therefore, efficiently inhibit several key steps of tumor metabolism.

Published results for the effects of ibuprofen on tumor cells are sparse, although ibuprofen is an equipotent COX-1 and COX-2 inhibitor [16,27] [28–30]. For glioma, documented effects have been published [9,31], although patients in these studies frequently used ibuprofen as pain reliever [32]. It was, therefore, of interest to also investigate the action of ibuprofen on glioma cells and to compare functional as well as metabolic effects of this NSAID to the well-described effects of diclofenac. In other tumor models, ibuprofen was shown to bind and activate the peroxisome proliferator-activated receptor γ (PPARγ) as observed from reporter gene assays [28]. It has been suggested that increased PPARγ suppresses cell proliferation of various tumor entities [29]. Additionally, ibuprofen (1–3 mM) is able to inhibit nuclear factor κ-light-chain-enhancer of activated B cells (NF-κB) activation by preventing the degradation of IκBα, the NF-κB inhibitory protein, in prostate cancer [30].

The NSAID diclofenac has been widely investigated and several of its effects are attractive for the treatment of tumors. In contrast, ibuprofen lacks investigation for its impact and effectiveness in glioma, and its molecular mechanisms of action are by-far less understood. Significant effects of ibuprofen on proliferation and migration in human cells could suggest the substance as adjuvant therapy with positive effects on glioma treatment. Therefore, the current study was designed to evaluate the anti-tumorigenic effects of ibuprofen in comparison to diclofenac. This analysis was conducted in concentrations of NSAIDs achievable in patients, and the effects of these compounds on proliferation, migration, and lactate formation of different human glioma cells are presented.

## Materials and Methods

### Ethics statement

The ethics board of the University of Regensburg, Germany, has approved the use of human material for this study (No° 11-103-0182). All patients provided written informed consent to participate in this study.

### Tumor cell lines

Human high-grade glioma cell lines U87MG and A172 were obtained from American Type Culture Collection (Manassas, USA). HTZ-349 is a primary tumor cell culture derived from resection of a human glioblastoma as previously described [33]. Tumor cells were maintained as monolayer cultures in Dulbecco’s Modified Eagles Medium (DMEM with 1g/L glucose; Sigma-Aldrich, Germany), supplemented with 10% fetal calf serum (FCS; Biochrom, Germany) at 37°C, 5% CO_2_, 95% humidity in a standard tissue culture incubator. For all functional assays, cells were starved by FCS deprivation (lactate measurements) or reduction to 5% (migration, proliferation, Western blot) 24 h prior experiments.

### Chemicals and drugs

Aspirin (ASA), ibuprofen-sodium, and diclofenac-sodium were purchased from Sigma-Aldrich, Germany. ASA and ibuprofen-sodium were dissolved in 1x PBS, and Diclofenac-sodium in DMSO. STATTIC was purchased from EMD Millipore, Germany. NaOxamat was purchased from Fluka, Germany and dissolved in media. Chemicals and drugs were further dissolved to final concentrations in media as shown in detail in the results and figure sections.

### Enzymatic determination of lactate in tumor cell supernatants

Lactate levels in cell culture supernatants were measured by means of a Cobas analyzer (Roche, Germany) using reagents from Roche (Germany) at the clinical laboratory of the Department of Neurology, University of Regensburg, Germany.

### LDH activity measurement

To control for LDH enzyme activity, the LDH-cytotoxicity assay (Promega, Mannheim, Germany) was used. The assay utilizes an enzymatic coupling reaction: LDH oxidizes lactate to generate NADH, which then reacts with pyruvate and a dye to generate yellow color. LDH activity was quantified with a plate reader (VarioSkan Flash Multimode Reader, Thermo Scientific, USA) at 490 nm absorption. Briefly, cells were seeded 24 h prior to treatment with 5 x10^3^ cells/well in serum free media and incubated with either the indicated concentrations of diclofenac (0.05–0.3 mM), ibuprofen (0.5–5 mM), or STATTIC (5–20 μM), NaOxamat (25 mM) was used as a positive control. 24 h later, LDH activity was measured.

### Determination of IC_50_ and cell proliferation

For determination of IC_50_ and cell proliferation, 2 x10^4^ cells of each cell line were incubated with either the indicated concentrations of diclofenac (0.05–0.2 mM), ibuprofen (0.5–2 mM), or ASA (0.05–0.2 mM) and corresponding controls before OD determination by crystal violet. Briefly, medium was exchanged with 0.5% crystal violet in 20% methanol solution and cells were stained for 10 min. After washing and drying, the crystal violet was diluted into a homogenous solution by addition of 0.1 M sodium citrate in 50% ethanol, and measured at 550 nm (VarioSkan Flash Multimode Reader, Thermo Scientific, USA). To determine IC_50_ concentrations, cells were treated for 24 h and proliferation analyzed at 0 and 96 h. Additionally, proliferation rates were determined at 0, 24, 48, 72, 96, and 120 h after onset of treatment. Proliferation rates with STATTIC (at concentrations of 5, 10, 20 μM) treatment were determined by use of the CyQuant Direct Cell Proliferation Assay according to the manufacturer’s protocol, higher STATTIC concentrations caused cell detachment. Additionally, the CyQuant assay served as a verification for the crystal violet assay. Briefly, cells were seeded and treated as described before. OD was measured at 0, 48, 96 and 144 h at 535 nm. For all assays, background fluorescence was subtracted and values were normalized to 0 h. Assays were performed in triplicates and repeated twice.

### Determination of cell migration

Tumor spheroids were generated by seeding 5 x 10^3^ cells onto agarose-coated wells (1% agarose in 1x PBS). Cells were cultured for 24 h to allow spheroid formation. Mature spheroids were transferred into non-coated 96-well plates containing the corresponding drug (diclofenac: 0.05, 0.1, 0.2 mM, ibuprofen: 0.5, 1, 2 mM, STATTIC: 5, 10, 15 μM). Cell migration was monitored at 0, 6, 24 and 30 h, taking into account the earliest time point when migration was measurable to prevent dilution of results by proliferation effects. The area covered by cells migrating away from the spheroid was photographed at indicated time points, and its greatest diameter was measured manually (ImageJ software, NIH, USA) by a investigator. Assays were performed in triplicates and repeated three times.

### Attachment assay

1.5 x 10^5^ HTZ-349 cells were seeded in 6-well plates and incubated for 24 h prior to treatment with 0.05 mM diclofenac or ibuprofen (0.1, 2 mM) alongside with the corresponding controls. After 24 h of treatment, attachment assays were performed with pre-treated cells as described earlier [34]. Briefly, 1.5 x 10^4^ cells were seeded in 96-well plates with treatment media. After 5, 15, 30, 60, and 120 min, wells were washed once with 1x PBS and attached cells were incubated for 6 h prior to quantification via CyQuant assay.

### Cell cycle analysis

For flow cytometric measurements of cellular DNA content, cells were fixed with ice-cold 70% methanol. After washing with PBS, cells were treated with 100 μg/ml RNase A (Invitrogen, Germany) for 20 min at 37°C. Subsequently, cells were stained with 50 μg/ml propidium iodide (Sigma-Aldrich, Germany) and analyzed with a FACS Canto™ (Becton Dickinson, Germany). Histograms were created using ModFit LT™ software (Verity Software House, Topsham, USA). At all times loss of cells was prevented by collecting also detached cells from the cell culture supernatant.

### Quantitative real-time PCR

For RNA isolation 1 x 10^5^ cells were seeded in 6-well plates, incubated for 24 h prior to treatment with 2 mM ibuprofen, 0.2 mM diclofenac, or 0.2 mM ASA and corresponding controls for 24 h. Total RNA was isolated by use of the Nucleo Spin RNA Plus Kit (Macherey-Nagel, Germany) according to the manufacturer’s instructions. Reverse transcription was performed with the Reverse Transcription System (Promega, Germany) according to the manufacturer’s protocol. Quantitative RT-PCR were performed as previously described [35].

### Western blot

To evaluate (phosphorylated) protein expression of LDH-A, STAT-3/pSTAT-3, and c-myc, whole cell lysates were prepared with RIPA-buffer and samples (40 μg) were subjected to Western blotting (10% SDS-PAGE). Membranes were sequentially analyzed with antibodies to GAPDH2 (Santa Cruz Biotechnology, Heidelberg, Germany), c-myc, LDH-A, STAT-3, phosphorylated STAT-3 (pSTAT-3) (all Cell Signaling, New England Biolabs GmbH, Germany), and β-actin (Sigma-Aldrich, Germany) in dry milk (1%). Expression was measured by chemo-luminescence (ECL Western Blot Bright, Biozym, Germany). Intensities of protein bands were measured with ImageJ software and protein regulation of 3 Western blots each (n = 3) was calculated by normalization to loading and treatment control using GraphPad Prism software (version 6, GraphPad Software, USA).

### Statistical analysis

Analyses of significant differences between treatment groups were performed by one- (lactate levels, LDH activity) or two-way ANOVA and in case of FACS analysis by Student´s t-test. Post-hoc tests were performed with p values < 0.05. Data were analyzed by GraphPad Prism software (version 6, GraphPad Software, USA). Confidence intervals (CI) were around 95%.

## Results

### The half maximal inhibitory concentrations (IC_50_) were similar for all glioma lines

**Fig 1:**
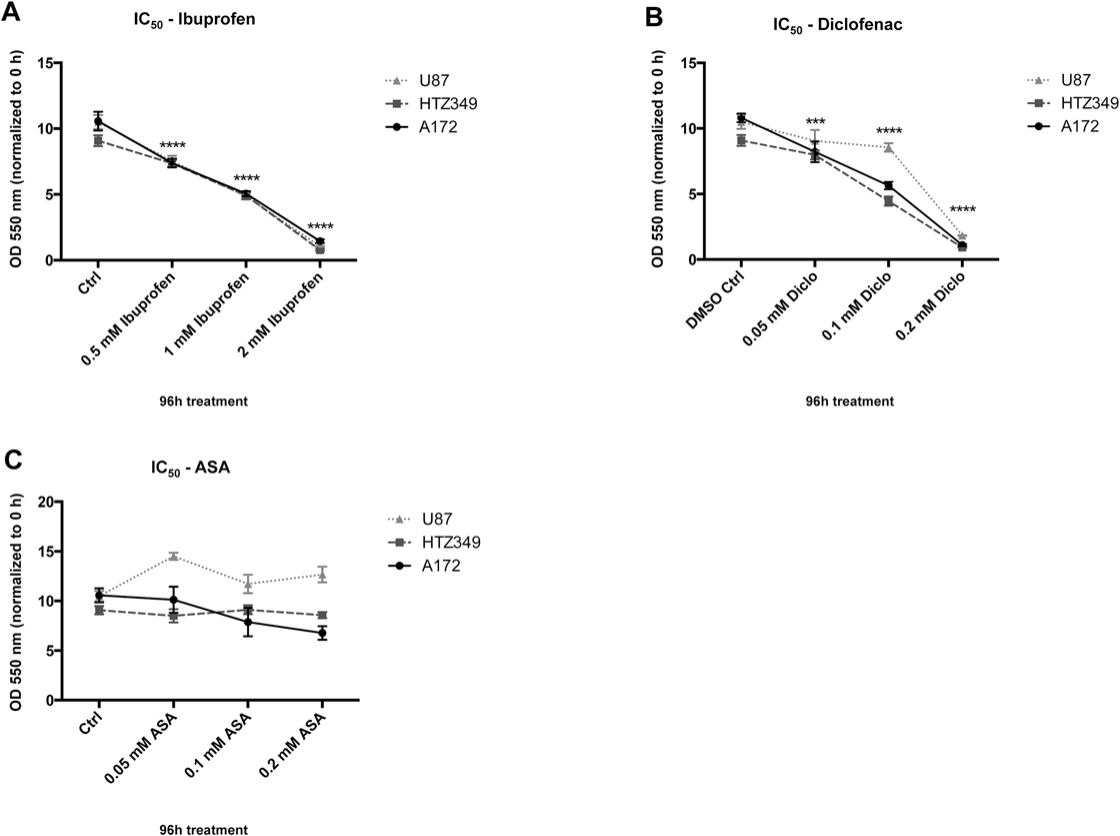
IC_50_ is distinct for diclofenac, ibuprofen, and ASA. Concentration-dependent cytotoxicity was investigated by treating the glioma cell lines HTZ-349, U87MG, and A172 in absence or presence of increasing concentrations of (A) ibuprofen (0.5–2.0 mM), (B) diclofenac (0.05–0.2 mM), or (C) ASA (0.05–0.2 mM) and DMSO as control in corresponding concentrations. The assays showed a significant linear concentration-dependent decrease of cell viability, which significantly differed between (A) ibuprofen (IC_50_, 1 mM) and (B) diclofenac (IC_50_, 0.1 mM). In contrast, (C) ASA had no significant effect on cell proliferation with given concentrations. Statistics: 95% CI, *** = 0.001 < p ≥ 0.0001, **** = p ≤ 0.0001.

First, non-toxic NSAID concentrations for further treatment of human malignant glioma cells were determined by culturing the human glioma cell lines HTZ-349, U87MG, and A172 in absence or presence of increasing concentrations of ibuprofen (0.5–2.0 mM; Fig 1A), diclofenac (0.05–0.2 mM; Fig 1B) or ASA (0.05–0.2 mM; Fig 1C) for 96 h. IC_50_ values were 1 mM for ibuprofen and 0.1 mM for diclofenac. ASA treatment had no significant effect on proliferation of HTZ-349, U87MG, or A172 glioma cells. The different IC_50_ of each substance did not vary substantially between the cell lines. For further experiments, we used concentrations ranging between 0.5 and 2 mM for ibuprofen and between 0.05 and 0.2 mM for diclofenac. ASA was used at the same concentrations as diclofenac, since both drugs are administered to patients at comparable concentrations when used on a long-term basis (e.g. secondary prevention of myocardial infarction and stroke).

### Ibuprofen and diclofenac decreased proliferation of glioma cells

**Fig 2:**
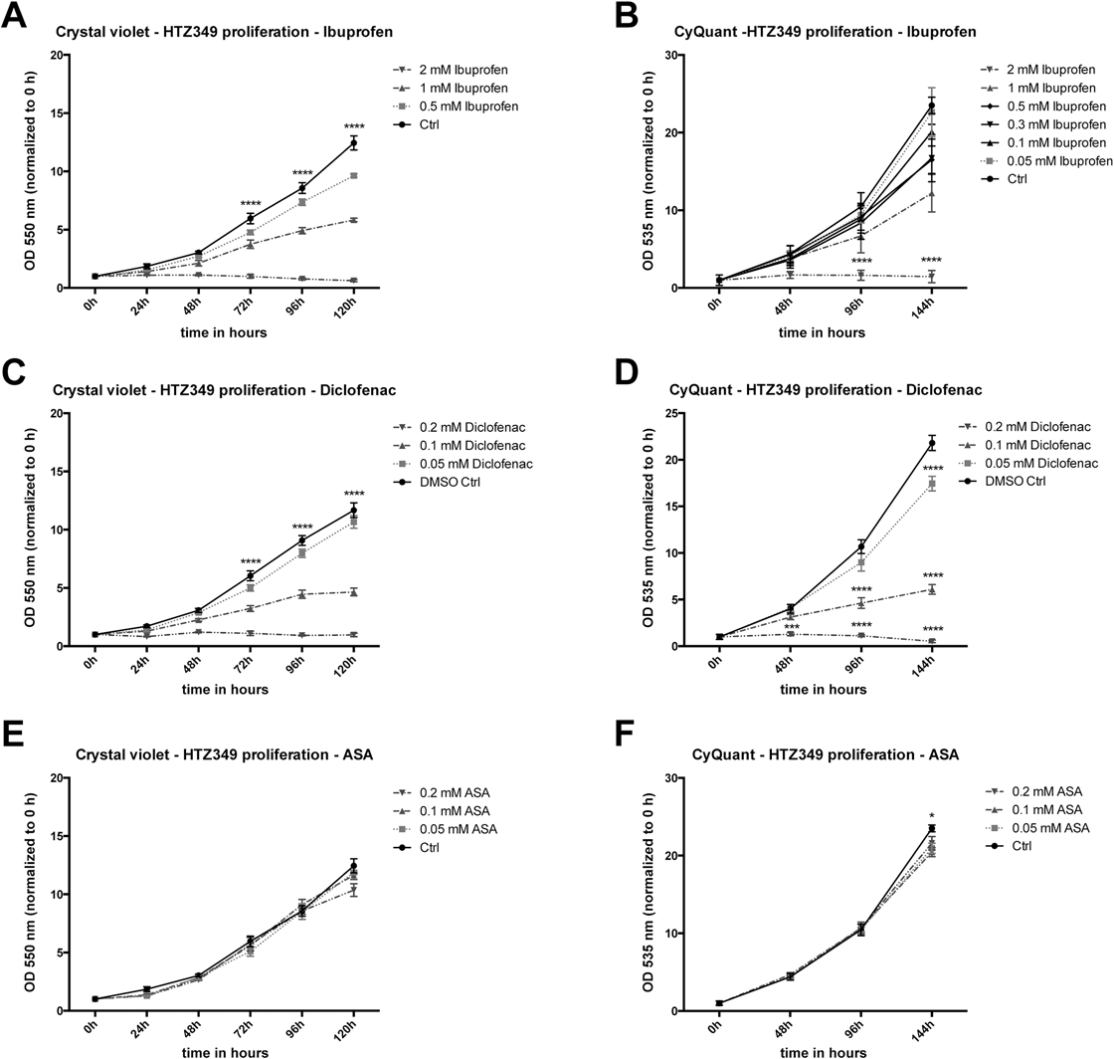
Ibuprofen and diclofenac decrease proliferation. Proliferation was analyzed using crystal violet staining and results were verified via the CyQuant Direct Cell Proliferation assay in HTZ-349. (A) Starting at 72 h, all ibuprofen concentrations (0.5, 1, 2 mM) resulted in significant reduction of HTZ-349 proliferation (compared to non-treated Ctrl, 95% CI, p < 0.05). 2 mM ibuprofen caused significant proliferation inhibition at 24 h (0.01 > p ≤ 0.001) and 48 h (p < 0.0001). (B) Proliferation decrease was verified in CyQuant measurements. Additionally, physiological ibuprofen concentrations (0.05–0.3 mM) were analyzed. Most demonstrated significant proliferation decrease after long-term treatment (144 h) compared to the non-treated controls (0.3–2 mM = p < 0.0001, 0.1 mM = 0.01 > p ≤ 0.001; 95% CI). (C) Similar proliferation reducing effects were obtained with diclofenac at respective concentrations (0.05, 0.1, 0.2 mM) (compared to DMSO control, 95% CI, p < 0.05). The highest concentration of 0.2 mM reached significant proliferation inhibition at 24 and 48 h compared to control (p < 0.0001). (D) CyQuant assay measurements of HTZ-349 treated with diclofenac were broadly in compliance with previously obtained results by crystal violet staining. (E) In contrast, ASA exhibited neither concentration- nor time-dependent effects, and only unspecific effects were observed at 96 h (0.05 mM: 0.05 > p ≤ 0.01) and 120 h (0.2 mM: p < 0.0001). (F) Corresponding CyQuant assay measurements of HTZ-349 treated with ASA verified the results. Treatment of A172 and U87MG cells showed similar results (see S1 Fig).

Proliferation inhibiting effects of the two NSAIDs, ibuprofen and diclofenac, on human glioma cell lines were analyzed in long-term assays (HTZ-349: Fig 2; A172, U87MG: S1 Fig). Daily crystal violet staining showed significantly impaired proliferation dependent on increasing dosages of ibuprofen (Fig 2A) and diclofenac (Fig 2C). Starting at 72 h, all dosages resulted in significant anti-proliferative effects (p ≤ 0.0001). Correspondingly, CyQuant measurements (B: ibuprofen, D: diclofenac) confirmed the crystal violet assays. Both NSAIDs had similar impact on proliferation of A172 (Figs A, C and E in S1 Fig) and U87MG (Figs B, D and F in S1 Fig). However, both NSAIDs were more effective on HTZ-349 and A172 than U87MG. Additional ibuprofen treatment (0.05, 0.1, 0.3 mM) using patient-relevant plasma concentrations of ibuprofen (0.1, 0.3 mM; FDA information: http://www.drugs.com/pro/ibuprofen.html) revealed that, on long-term exposure, (144 h) low ibuprofen concentrations of ≤ 0.1 mM were efficient to cause a significant proliferation decrease in HTZ-349. Proliferation remained generally unaffected by ASA (HTZ-349: Figs E and F in Fig 2, A172: S1E Fig). Only U87MG responded with a decrease after 120 h (S1F Fig). Thus, ASA was used as control, but not further investigated for tumor cell inhibiting capacities.

### Ibuprofen led to G1/G0 cell cycle arrest whereas diclofenac caused an arrest at G2/M

**Fig 3:**
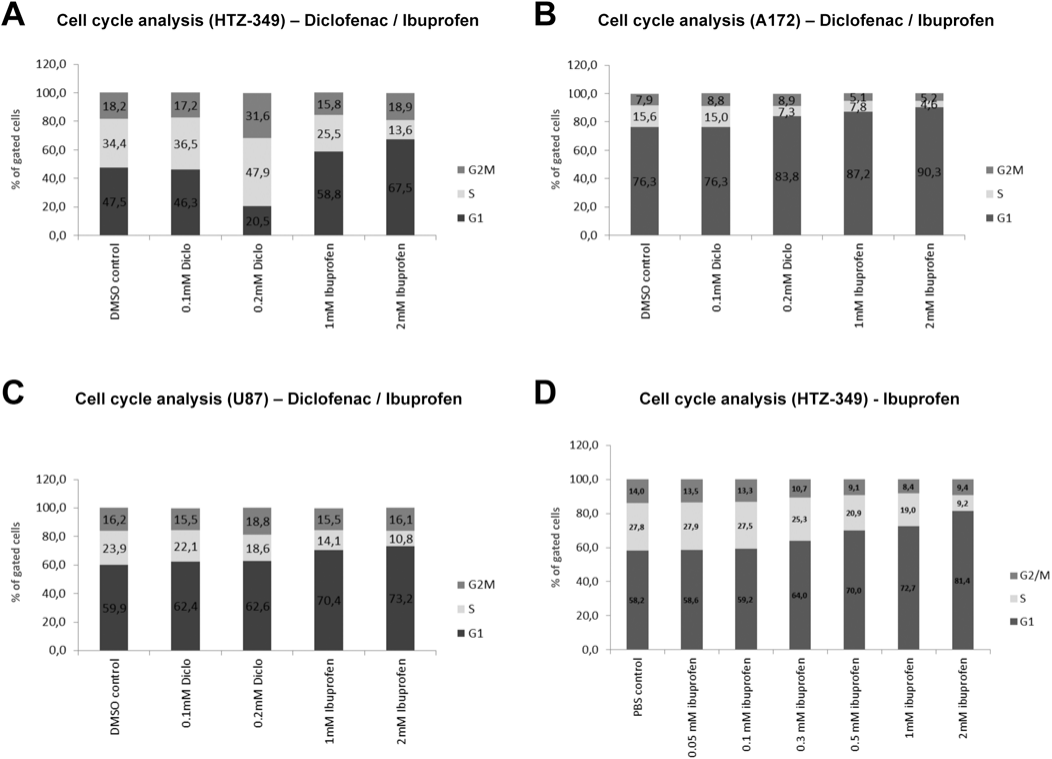
Ibuprofen and diclofenac led to cell cycle arrest at different checkpoints. Human glioma cell lines HTZ-349 (A), A172 (B), and U87MG (C) were cultured in the presence of increasing concentrations of diclofenac (0.1, 0.2 mM) or ibuprofen (1, 2 mM) and respective DMSO controls. Cells were harvested for cell cycle analysis after 48 h of incubation. Proliferation was significantly reduced as measured by a reduced number of cells in S-phase in all conditions except 0.1 mM diclofenac in A172 as well as 0.1 and 0.2 mM diclofenac in HTZ-349. With diclofenac treatment, HTZ-349 and U87MG cells showed an accumulation of cells in the G2/M (U87MG) and S-phase (HTZ-349), whereas A172 arrested in the G1 phase. Ibuprofen generated accumulation of cells in the G1 phase in all cell lines. Additional treatment with 0.05–2 mM ibuprofen (D) confirmed dose-dependent accumulation of HTZ-349 in G1 accompanied by a decrease of cells in S- and G2/M-phase. Bar graphs show mean values of three independent experiments. Histograms are depicted in the supplements (S2 Fig) and show representative plots of each treatment condition.

Cell cycle analyses were performed to distinguish the individual effects of ibuprofen or diclofenac treatment on glioma cell proliferation and to confirm the observed effects were not caused by toxicity of either NSAID (Fig 3). Human glioma cell lines HTZ-349 (Fig 3A), A172 (Fig 3B), and U87MG (Fig 3C) were cultured in the presence of increasing concentrations of ibuprofen (1, 2 mM) or diclofenac (0.1, 0.2 mM) as well as corresponding controls. After 48 h, all cells were harvested for cell cycle analysis by flow cytometry.

Ibuprofen treatment resulted in a concentration-dependent accumulation of cells in G1/G0 phase in all cell lines (HTZ349: 1 mM ibuprofen: p = 0.001; 2 mM ibuprofen: p = 0,0001; A172: 1 mM ibuprofen: p = 3.8 x 10^−6^; 2 mM ibuprofen: p = 6.9 x 10^−7^; U87MG: 1 mM ibuprofen: p = 4.7 x 10^−5^; 2 mM ibuprofen: p = 0.0006) concomitant with a decrease of cells in S-phase (HTZ349: 1 mM ibuprofen: p = 0.0007; 2 mM ibuprofen: p = 1.2 x 10^−6^; A172: 1 mM ibuprofen: p = 0.0002; 2 mM ibuprofen: p = 0.0006; U87MG: 1 mM ibuprofen: p = 8.9 x 10^−5^; 2 mM ibuprofen: p = 0.0002), indicating impaired proliferation.

In contrast, diclofenac had different effects on each cell line. The amount of cells in S-phase was decreased in A172 and U87MG (A172: 0.1 mM diclofenac: p = 0.21; 0.2 mM diclofenac: p = 0.0001; U87MG: 0.1 mM diclofenac: p = 0.03; 0.2 mM diclofenac: p = 0.002). However, HTZ-349 cells accumulated in S- and G2/M-phase with increasing concentrations (HTZ349: 0.1 mM diclofenac: p = 0.009; 0.2 mM diclofenac: p = 0.002). Here, cell cycle arrest was accompanied by the appearance of a sub-G1 peak along with cellular debris, indicating cellular disintegration and possibly cytotoxic effects (S2A Fig). This was not observed with ibuprofen treatment or diclofenac in the other cell lines. However, whereas HTZ-349 and U87MG cells accumulated in G2/M and S-phase, A172 cells arrested in G1/G0 similar to ibuprofen conditions. Physiological ibuprofen concentrations had long-term inhibiting effects on proliferation (Fig 2B) and caused cell cycle alterations already after 48 h (Fig 3D). Dose-dependent accumulation of cells in G1 was observed for ibuprofen (0.3 mM: p = 4.2 x 10^−6^; 0.5 mM: p = 1.2 x 10^−5^; 1 mM: p = 3.3 x 10^−8^; 2 mM: p = 7.1 x 10^−5^). A concomitant shift of cells from the S-phase (0.3 mM: p = 0.01; 0.5 mM: p = 0.003; 1 mM: p = 9.7 x 10^−7^; 2 mM: p = 8.7 x 10^−6^) resulted in decreased proliferation. Detailed FACS analysis plots for every condition and cell line are shown in the supplements (HTZ-349: Figs A and D in S2 Fig, A172: S2B Fig, U87MG: S2C Fig).

### Ibuprofen and diclofenac decreased migration of glioma cell lines

**Fig 4:**
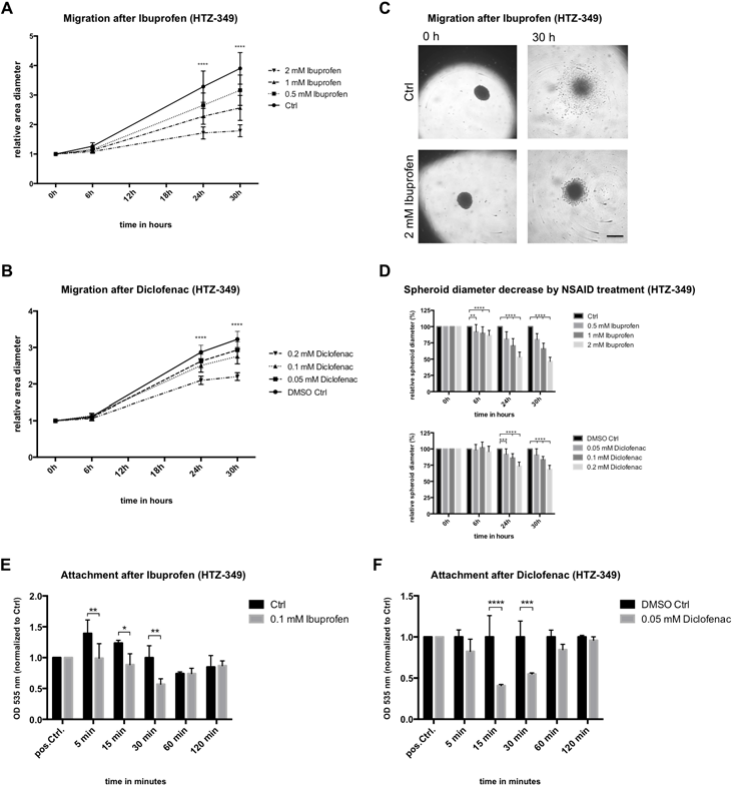
Ibuprofen and diclofenac decrease migration. Spheroids of human glioma cell line HTZ-349 (5 x 10^3^ cells/well) were cultured in presence of increasing (A) ibuprofen (0.5–2 mM) or (B) diclofenac concentrations (0.05–0.2mM) and monitored for 30 h. During this time, pictures of cells migrating from the spheroids were taken periodically to monitor migratory capacity at treatment conditions (C) (scale bar = 500 μm). Migration was significantly inhibited by ibuprofen (A) in a time- and concentration-dependent manner. After 24 h, all concentrations demonstrated significantly greater migration over controls (95% CI, **** = p < 0.0001). (D) A high concentration of ibuprofen, 2 mM, resulted in significant migration inhibition noticeable by 6 h (S3A Fig). Diclofenac resulted in similar (B), although less pronounced (D), effects. Measurement of attachment capacities after 0.1 mM ibuprofen (E) or 0.05 mM diclofenac (F) revealed adhesion deficits of HTZ-349 cells compared to controls within the first 30 minutes (95% CI, * = 0.05 > p ≤ 0.01, ** = 0.01 > p ≤ 0.001, *** = 0.001 > p ≤ 0.0001, **** = p < 0.0001.).

Glioma pathogenesis is characterized by rapid tumor cell migration. Former results [35] have shown that lactate influences migration of human glioma cell lines. Here, influence on glioma cell migration was investigated by spheroid migration assays (Fig 4). Spheroids of glioma cells were transferred, to be cultured in presence of increasing ibuprofen (0.5 to 2 mM) or diclofenac (0.05 to 0.2 mM) concentrations, and cells were given time to migrate from the spheroid (Fig 4C) for up to 30 h. Strong inhibitory effects on migration were obtained with all ibuprofen concentrations in HTZ-349 (Fig 4A and S3A Fig). Starting 24 h after treatment, concentration- and time-dependent migration-inhibiting effects were observed. The same applied to A172 (S3B Fig) and U87MG (S3C Fig), which responded significantly to ibuprofen starting 6 h after treatment. Additionally, all diclofenac concentrations significantly decreased migration starting at 24 h in a dose-dependent manner (Fig 4B and S3D Fig). Similar results were obtained for A172 with diclofenac (S3E Fig), whereas U87MG were—in contrast to ibuprofen treatment (S3C Fig)—resistant to diclofenac until 30 h of treatment (S3F Fig).

Ibuprofen proved to be significantly more effective to reduce the migratory capacity in all glioma cells (Fig 4D). 30 h after exposure to 2 mM ibuprofen, spheroid diameters were only 45% of the control size, whereas in the corresponding dicofenac treated (0.2 mM) spheroids diameters were 68% of the control. The most prominent effects were achieved in U87MG cells, where ibuprofen treatment resulted in a spheroid diameter decrease of 40%, whereas diclofenac inhibited spheroid migration only by 11% (S3C and S3F Fig).

The spheroid assay is not designed to exclude confounding effects that may arise from impaired proliferation. However, the observed reduction of migratory capacity is likely independent, since significant reduction of proliferation was measurable only from 48 h on, as shown above (Fig 2 and S1 Fig).

Investigation of mechanisms underlying reduced migratory capacity revealed decreased attachment of HTZ-349 cells treated with low dosages of either ibuprofen (Fig 4E) or diclofenac (Fig 4F) until 30 minutes after seeding. To gain deeper insight into whether the cytoskeleton may be affected by NSAID treatment, resulting in the observed migration deficits, we performed phalloidin staining of actin filaments (S4 Fig). Significantly larger cells with enhanced spreading were observed with higher dosages of diclofenac (Figs A and B in S4 Fig) or ibuprofen (Figs C and D in S4 Fig). We assumed an increase of filamentous actin (F-actin) was responsible for migration deficits and attachment delay. However, measurement of F-actin and G-actin (globular actin) ratios revealed neither significant increase nor decrease of F-actin levels (Figs E, F and G in S4 Fig).

### Ibuprofen and diclofenac inhibited LDH activity and reduced lactate production

**Fig 5:**
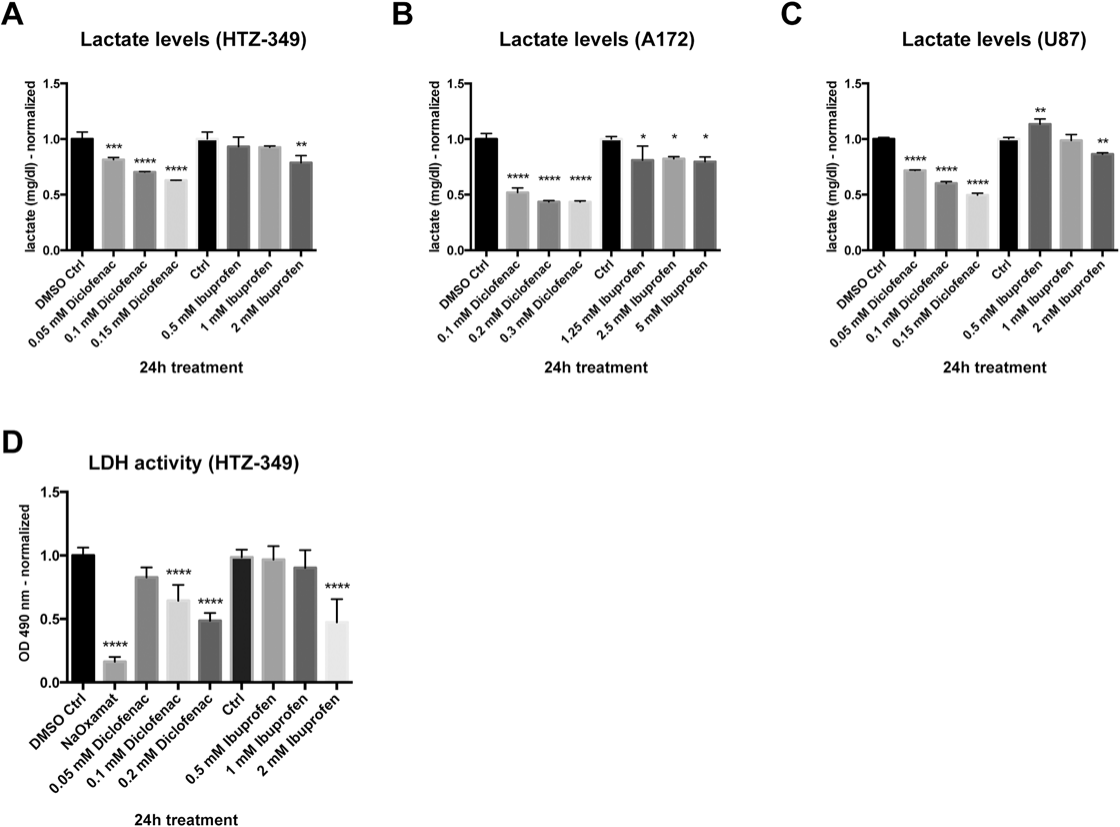
Ibuprofen and diclofenac decrease lactate levels and LDH activity. Human glioma cell lines HTZ-349, A172, and U87MG (10^5^ cells/well) were cultured for 24 h with increasing concentrations of ibuprofen or diclofenac as indicated, then supernatants were harvested for lactate level measurement (A–C). Measurements were normalized to cell number. Diclofenac treatment resulted in a significant decrease of lactate at all concentrations and in all cell lines. In contrast, ibuprofen cause a reduction of lactate accumulation only at concentrations of 2 mM in HTZ-349 and U87MG. These observations are reflected by LDH activity measurements (D) which exhibited decreased activity with increasing concentrations of diclofenac. LDH activity reduction with ibuprofen treatment occurred only at a concentration of 2 mM. Statistics: 95% CI, * = 0.05 > p ≤ 0.01, ** = 0.01 > p ≤ 0.001, *** = 0.001 > p ≤ 0.0001, **** = p < 0.0001.

Recently, our group demonstrated that diclofenac treatment causes decreases of lactate production in the mouse glioma cell line GL261 [18]. It was, therefore, investigated whether ibuprofen had a similar effect on lactate concentration and LDH activity. To investigate this, all cell lines were treated with ibuprofen (0.5, 1.0, and 2.0 mM) and diclofenac (0.05, 0.1, and 0.15 mM) (Fig 5). Supernatants were analyzed for extracellular lactate concentrations after 24 h when proliferation was not yet altered by the treatment (Fig 2 and S1 Fig). With ibuprofen, lactate production was significantly reduced only with the highest concentration of 2 mM ibuprofen in HTZ-349 and U87MG glioma cells (Fig 5A and 5C) (p < 0.05). In A172, ibuprofen did not substantially reduce extracellular lactate levels in low or in high concentrations (Fig 5B). Diclofenac significantly reduced lactate levels in a dose-dependent manner in all cell lines and the effects were stronger than those observed with ibuprofen (Figs A–C in Fig 5). Along with decreased lactate levels, LDH activity was inhibited after treatment with 0.1 or 0.2 mM diclofenac as well as with 2 mM ibuprofen (Fig 5D). Sodium oxamate, a competitive inhibitor of LDH, was used as a positive control.

### Ibuprofen and diclofenac decreased STAT-3 phosphorylation, but modulated c-myc differently

**Fig 6:**
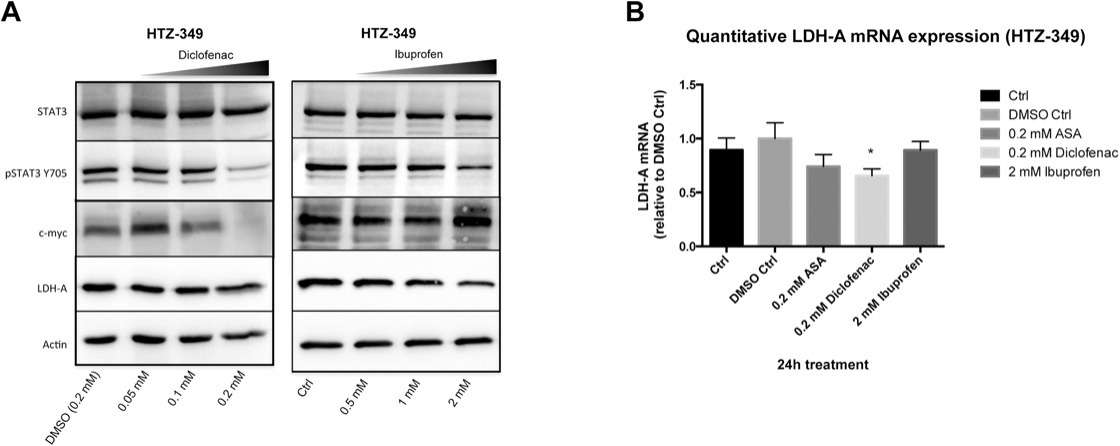
Ibuprofen and diclofenac affect STAT-3 signalling differentially. Transcription factors within the STAT-3 signalling pathway were investigated by Western blot (40 μg of protein) after incubation with increasing ibuprofen (0.5, 1, 2 mM) or diclofenac concentrations (0.05, 0.1, 0.2 mM) for 24 h. (A) Ibuprofen and diclofenac reduced STAT-3 phosphorylation in HTZ-349 cells without affecting total STAT-3 levels (for detailed quantification see S5 Fig). The STAT-3 downstream target c-myc was significantly upregulated by ibuprofen (S5A Fig), whereas diclofenac blocked pSTAT-3 and c-myc in a concentration-dependent manner (S5B Fig). LDH-A, a direct target of c-myc, showed negative trends, but not to significant extent. Corresponding quantitative RT-PCR revealed significant LDH-A transcript decrease with diclofenac (0.2 mM, compared to control and DMSO control, 95% CI, * = 0.05 > p ≤ 0.01). Ibuprofen (2 mM) had no impact on LDH-A expression (B). Similar observations were made with ASA (0.2 mM). Corresponding results for A172 and U87MG were generated (see S6 and S7 Figs).

Many highly proliferative solid tumors are characterized by intrinsic or induced expression of transcription factors which affect the cell cycle. High expression and activation of STAT-3 and overexpression of its downstream target c-myc are well known to be pathophysiological mechanisms in malignant glioma [36]. As diclofenac impacts LDH-A, which is targeted directly by c-myc [37], STAT-3 and c-myc were investigated as possible modulators of the observed functional effects. Protein expression levels and phosphorylation of c-myc, pSTAT-3, STAT-3, and LDH-A in HTZ-349 (Fig 6A and S5 Fig), A172 (Figs A and B in S6 Fig), and U87MG (Figs A and B in S7 Fig) were determined after 24 h exposure to increasing ibuprofen or diclofenac concentrations. As shown in Fig 6A, increasing concentrations of both ibuprofen and diclofenac led to a reduction of STAT-3 phosphorylation without affecting total STAT-3 expression in HTZ-349, this was also observed in A172 (S6 Fig) and U87MG (S7 Fig). Against expectation, c-myc expression was enhanced significantly after treatment with ibuprofen, whereas diclofenac reduced c-myc. Quantifications by Western blot replicate assays revealed these effects to be consistent (see S5, S6B and S7B Figs). However, it has to be noted that changes of pSTAT-3 and c-myc expression were significant only with high ibuprofen treatment (2 mM) and remained unchanged at physiological concentrations (Figs C and D in S5 Fig). This inducing effect of ibuprofen was consistently observed in all cell lines (Fig 6A, S5A Fig; Figs A and B in S6 and S7A Figs), whereas diclofenac acted contrarily in HTZ-349 and A172 (Fig 6A, S5B Fig; Figs A and B in S6 Fig). Combined with the decrease of STAT-3 phosphorylation, LDH-A revealed a trend towards lower expression at higher concentrations of both agents. As LDH-A persists with a long protein half-life [38], mRNA expression was assessed via qRT-PCR after 24 h exposure of the cells either to ibuprofen, diclofenac, or ASA. For HTZ-349 (Fig 6B) and U87MG (Figs B and C in S7 Fig), a significant decrease of LDH-A was confirmed after exposure to diclofenac, and for A172, a consistent downward trend was detected (S6C Fig). In contrast, LDH-A expression was not decreased in either HTZ-349 (Fig 6B and S5 Fig) or in U87MG (S7 Fig) cells following ibuprofen treatment, while down-regulation was significant in A172 cells (S6C Fig).

### Restricting phosphorylation of STAT-3 led to a decrease of c-myc and LDH-A as well as reduced proliferation and migration

**Fig 7:**
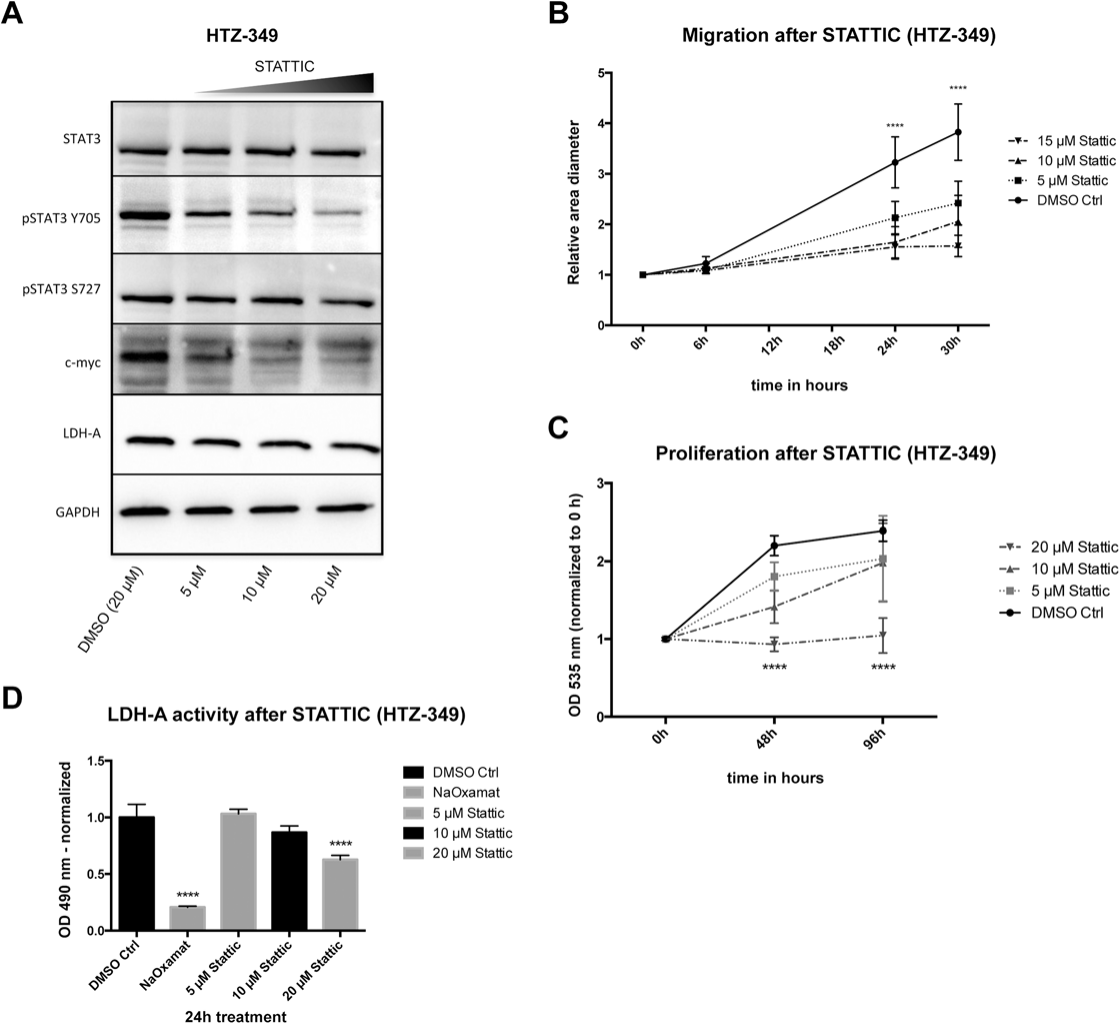
Inhibition of STAT-3 phosphorylation modulates signalling along with functional effects. STAT-3 phosphorylation at Y705 is inhibited by STATTIC. HTZ-349 cells were exposed to increasing STATTIC concentrations (5, 10, 15/20 μM) for 24 h. (A) pSTAT-3, c-myc, and LDH-A expression were investigated by Western blot (40 μg of protein). A decrease in both phosphorylation of pSTAT-3 and expression of its downstream targets, c-myc and LDH-A, was observed. Total STAT-3 and STAT-3 phosphorylated at the S727 residue remained unchanged (for quantification see S8A Fig). Migration (B) and proliferation (C) showed a significant concentration-dependent inhibition (95% CI, **** = p < 0.0001) with increasing doses of STATTIC. Migration was inhibited significantly from 24 h on with all concentrations (B), whereas proliferation was reduced significantly with 20 μM starting at 48 h (C). LDH-A activity was reduced in a concentration-dependent way, with significance when cells were exposed to 20 μM of STATTIC (D) (95% CI, **** = p < 0.0001).

To substantiate a direct effect of ibuprofen and diclofenac on STAT-3 phosphorylation, the specific STAT-3 inhibitor STATTIC was used. STATTIC restricts the phosphorylation of STAT-3 at the physiologically relevant tyrosine residue 705 (Y705) [39]. Treatment significantly decreased STAT-3 phosphorylation in a concentration-dependent manner, along with reduction of the downstream target c-myc (HTZ-349: Fig 7A and S8A Fig; A172: S9A Fig; U87MG: S10A Fig). STATTIC exerts a specific effect on Y705 and total STAT-3 and STAT-3 phosphorylated at the serine residue 727 (S727) remained constant after 24 h treatment. LDH-A expression was not affected, except in U87MG cells which exhibited a significant decrease in LDH-A at 20 μM STATTIC compared to DMSO Ctrl (0.05 < p ≤ 0.01). LDH-A activity was reduced significantly after 24 h of treatment in these cells (p < 0.0001), suggesting a functional effect. Additionally, qRT-PCR revealed a strong decrease of LDH-A mRNA expression 24 h after treatment with 20 μM STATTIC (S8B Fig). In human glioma cells exposed to STATTIC, migration (HTZ-349: Fig 7B; A172: S9B Fig; U87MG: S10B Fig) and proliferation (HTZ-349: Fig 7C; A172: S9C Fig; U87MG: S10C Fig) decreased considerably, depending on increasing concentrations. STATTIC was observed to decrease migration similarly to ibuprofen in HTZ-349 (Fig 4D and S8C Fig), A172 (S3B and S9B Figs), and U87MG cells (S3C and S10B Figs).

## Discussion

In this study, the effects of ibuprofen and diclofenac on proliferation, migration and lactate formation of human HTZ-349, U87MG and A172 glioma cell lines were investigated. In addition, the functional consequences and molecular mechanisms of these effects were explored. Our data provide evidence that long-term treatment with ibuprofen reduces human glioma cell proliferation and migration ability more effectively than diclofenac. Our results indicate that both agents are significantly more effective than ASA, which caused no significant changes to human glioma cells in any assay investigated here. Since anti-tumorigenic effects of ASA are discussed in the literature [16], the missing effect here is probably due to the fact that ASA was intentionally used at concentrations normally for long term administration to patients rather than acute treatment. Diclofenac was applied in physiological concentrations which correspond to serum levels of approved therapeutic use in humans (FDA information for diclofenac: http://www.drugs.com/pro/diclofenac.html). In contrast, we observed most significant effects of ibuprofen in our *in vitro* assays with concentrations exceeding the appropriate physiological concentrations (FDA information for ibuprofen: http://www.drugs.com/pro/ibuprofen.html). Long-term treatment in routinely recommended concentrations has promising inhibitory effects on cell cycle and cell proliferation, in addition, higher ibuprofen concentrations may be reached in regions of blood-brain-barrier disruption which can accompany glioma [40].

A dose-dependent highly-significant restriction of cell migration and proliferation by ibuprofen, and to a lesser extent, diclofenac, was observed. Ibuprofen was determined to be a strong and potent inhibitor of the cell cycle at the G1/G0 state in all cell lines tested, whereas diclofenac arrested them mainly at the G2/M check-point and in S-phase. This corresponds well to published literature, where a G1/G0 arrest by ibuprofen has been shown in colon [41] and prostate cancer cells [42]. In contrast, diclofenac treatment led to an accumulation of cells in S- and G2/M phase. This is in line with our previous results regarding glioma cells [18] as well as data from other tumor entities [43]. The sub-G1 peak observed in HTZ-349 probably also indicates cytotoxic effects, even though a contribution of apoptotic cell death cannot be fully excluded. However, this effect was not observed in any other cell line. More insight into the differential sensitivities of glioma cells towards NSAIDs regarding cell cycle arrest could further elucidate their tumor inhibiting abilities.

To explain the data obtained in this work, we hypothesized a potential COX-independent mechanism for the non-selective COX inhibitor ibuprofen [27], as ASA, a preferential COX-1 inhibitor [27], exhibited no effects, and ibuprofen non-selectively induced more significant effects than diclofenac, a slightly preferential COX-2 inhibitor [15]. Missing effects of ASA are likely due to our use of concentrations administered to patients on a long-term basis and correspond to a controversial debate within published literature [16], where often only dosages of acute treatment are successful. Both COX-dependent [44] and -independent mechanisms for anti-tumorigenic action of NSAIDs have been reported, and merging studies stress other modes of action for NSAIDs besides COX-inhibition. Results of rat model experiments suggest that NSAIDs, including ibuprofen, may prevent colon carcinogenesis via induction of enzymes which detoxify potential carcinogens [45]. In addition, diclofenac induces apoptosis partly by prevention of tumor necrosis factor α (TNF-α) induced nuclear translocation of NF-κB [46]. However, TNF-α signalling is not the main contributor to apoptosis induction, supporting a synergistic action between TNF-α and further inhibitory properties of diclofenac [46].

Reduced cell migration capacity and delayed attachment may be caused by a disrupted homeostasis of the cytoskeleton due to ibuprofen or diclofenac treatment. Indeed, phalloidin staining of actin filaments revealed a change in morphology after treatment. After NSAID treatment, cells spread more and were thus significantly larger in size. Continuous actin polymerization at the leading edge is required to stabilize membrane protrusions and enable cell migration [47]. We hypothesized a treatment induced increase of F-actin which exceeds the homeostasis between actin states consequently interfering with migration [48]. However, our data did not reveal significant changes in the F-/G-actin ratio of treated cells. This indicates that other cytoskeletal components such as the microtubule network may be affected, as those also contribute to establish and maintain polarity in migrating cells [49,50]. However, these investigations were beyond the scope of this work.

In our assays, NSAID treatment induced attachment delay, but increased adherence at the end of the assays, which was indicated by enhanced spreading. Cell adhesion formation and maintenance is a very complex and well-regulated process (for detailed reviews see [51,52]). Hence, at this point, we can only speculate about a possible role of ibuprofen and diclofenac in one or multiple mechanisms mediating the proper turnover of cell attachment. Future studies will unravel the impact of ibuprofen and diclofenac treatment on the above mentioned aspects of adhesion formation, regulation, and it´s integration into different migration steps.

Our data further show that ibuprofen reduces lactate levels, but to a lower extent than diclofenac. The results obtained here on lactate levels following diclofenac treatment are in accordance with earlier data from our group [35], where we showed that diclofenac reduced extracellular lactate levels and lead to a significant reduction of glioma cell migration. The decrease of lactate was induced at physiological, non-toxic concentrations of diclofenac. This is in support of published data from our group, which indicated that reduced lactate levels are accompanied by a dose-dependent inhibition of cell growth [17,18]. Consequently, it can be assumed that the effects of ibuprofen on migration and proliferation of glioma cells are less dependent on lactate modulation, especially at lower dosages.

A substantial modulation of STAT-3 signalling by ibuprofen and diclofenac was assumed as LDH-A is a direct target of c-myc [37], which itself is induced by STAT-3 [53]. Indeed, both agents decreased STAT-3 phosphorylation at its physiological active amino acid Y705. STAT-3 is a pivotal transcription factor, phosphorylated by receptor-associated kinases in response to several stimuli, and thus is a key player in many cellular processes such as cell growth and apoptosis. STAT-3 has been assumed to modulate the transition from an epithelial to a migratory mesenchymal phenotype in glioblastoma [26] and to promote tumorigenicity of glioma stem-like cells [54]. Modulation of STAT-3 by ibuprofen or diclofenac may therefore constitute a mechanism of action that could improve the treatment of high-grade glioma. To further substantiate the role of STAT-3, we investigated the signalling pattern and functional effects on proliferation and migration after treatment with STATTIC, a specific inhibitor of STAT-3 phosphorylation. As expected, and similar to the effects of diclofenac and ibuprofen, STATTIC restricted STAT-3 phosphorylation, leading to cell cytotoxicity at high levels [39] along with a down-regulation of LDH-A activity [55,56]. This is in line with published data showing anti-tumor activity of STATTIC and its induction of chemo- and radio-sensitivity [57]. However, despite its effect on LDH-A expression and activity, ibuprofen led to an increase of c-myc protein expression.

Ibuprofen may, therefore, exert a c-myc-independent mechanism of LDH-A activity modulation, which occurs only with the highest concentrations of ibuprofen, and in congruence with the decrease of lactate levels. This may be due to a reduced cell number or cytotoxicity, even though data were normalized to cell number and ibuprofen did not influence proliferation within 24 hours. Ibuprofen may consequently act via inhibition of NF-κB, mediated by suppression of TNF-induced IκB kinase activity, which prevents IκBα degradation [58]. In addition, although not directly proven, restriction of STAT-3 phosphorylation could constitute a c-myc- and COX-independent mechanism of the anti-tumorigenic effect of ibuprofen and diclofenac. Ibuprofen decreased migration as effectively as STATTIC, whereas diclofenac was less effective, indicating a stronger effect of ibuprofen on STAT-3. However, both NSAIDs achieved better restriction of proliferation than the STAT-3 specific inhibitor STATTIC, indicating additional (diclofenac) or alternative (ibuprofen) mechanisms.

In addition to inhibition of LDH expression and activity mediated by STAT-3 and c-myc, altered lactate efflux may be an alternative mechanism which could account for the observed reduced lactate levels after treatment with diclofenac and ibuprofen. Lactate is transported through the plasma membrane by proton-linked monocarboxylate transporters [23]. NSAIDs may possess monocarboxylic acid structures and may, therefore, competitively inhibit MCT function [59–61]. Glioma cells mainly express MCT-1 and MCT-4, but with differences in their cytosolic or membrane-bound localization [62], hence both agents influence additional pathways.

New anti-neoplastic candidates derived from drug-repurposing studies often yield their effects only in concentrations far above those that are regularly reached in humans, which is a crucial point for translation into clinical trails. Therefore, the significant glioma cell restricting effects of ibuprofen in high dosages might be expected, but more importantly, also physiological dosages were sufficient to inhibit glioma cells, when administered on a long-term basis. To further improve glioma treatment, a combined therapy with ibuprofen and diclofenac may be of value. Additionally, localized increases in ibuprofen concentration in regions of interest may be possible by use of ibuprofen nanocarriers [40].

In conclusion, this study indicates a novel mechanism for the functional effects yielded by ibuprofen in glioma cell lines *in vitro*. Our data further suggest the need to evaluate the response to NSAIDs and their efficacy as an adjuvant therapy in patients with high-grade glioma, especially glioblastoma. NSAIDs are clinically available, reach therapeutic concentrations in humans, and are well tolerated. Thus, clinical trials could integrate ibuprofen or diclofenac into a standard therapy regimen with the aim to restrict glioma proliferation and migration. Further comparative studies should affirm the effects of ibuprofen and diclofenac on glioma cells *in vitro* and *in vivo* as well as elucidate their molecular mechanisms of action in more detail.

## Supporting Information

S1 Fig Both Ibuprofen and diclofenac decrease proliferation in A172 and U87MG. Corresponding to Fig 2, the proliferation abilities of glioma cell lines A172 and U87MG were analyzed after ibuprofen (0.5, 1, 2 mM), diclofenac (0.05, 0.1, 0.2 mM), or ASA (0.05, 0.1, 0.2 mM) treatment (A, B). At 96 and 120 h, all ibuprofen concentrations achieved significant reduction of A172’s and U87MG’s proliferation (compared to non-treated Ctrl, 95% CI, p < 0.0001). Ibuprofen proved to be more effective in A172 (A). Significant values were as follows: (A) 48 h: 2 mM = 0.001 > p ≤ 0.0001, 48 h: 1 mM = 0.05 > p ≤ 0.01; (B) 48 and 72 h: 2 mM = p < 0.0001, 72 h: 1 mM = 0.001 > p ≤ 0.0001. (C, D) Similar proliferation inhibiting effects were obtained with diclofenac. At 96 and 120 h, all concentrations resulted in a significant reduction (compared to DMSO Ctrl, 95% CI, p < 0.0001). However, diclofenac was not as effective as ibuprofen on A172 cells (compare Figs A and C). Significant values were: (C) 48 and 72 h: 2 mM = p < 0.0001, 72 h: 1 mM = p < 0.0001; (D) 48 h: 0.2 mM = 0.01 > p ≤ 0.001, 72 h: 2 mM = p < 0.0001. (E) ASA had time-dependent effects in A172 with the highest concentration of 0.2 mM (72, 96 and 120 h: 0.2 mM = 0.001 > p ≤ 0.0001), but was not as effective as diclofenac or ibuprofen. (F) ASA has neither concentration- nor time-dependent effects on U87MG cell proliferation as all ASA concentrations significantly decrease proliferation only at 120 h (significant value: 96 h: 2 mM = 0.01 > p ≤ 0.001).
(TIF)

S2 Fig Ibuprofen and diclofenac induce cell cycle arrest in HTZ-349 A172 and U87MG. Ibuprofen and diclofenac induced cell cycle arrest in all cell lines, although at different checkpoints. The most prominent effects were observed from diclofenac treatment in HTZ-349, where increasing concentrations resulted in a sub-G1 peak, indicating cell death (Figs A and D). This was not observed in A172 (Fig B) or U87MG (Fig C). Figures depict representative histograms of each treatment.
(TIF)

S3 Fig Ibuprofen reduces migration in HTZ-349, A172 and U87MG. Ibuprofen decreased migration in a time- and concentration-dependent manner in all glioma lines starting 6 h after treatment compared to a non-treated control (95% CI, **** = p < 0.0001). (A) Bar charts corresponding to the migration curves for HTZ-349 as shown in Fig 4A. (B) Similar response to ibuprofen was observed for the glioma line A172. (C) Response was increased in U87MG cells as all concentrations achieved significant inhibition of migration after only 6 h of exposure to ibuprofen. Statistics: * = 0.05 > p ≤ 0.01, ** = 0.01 > p ≤ 0.001, *** = 0.001 > p ≤ 0.0001, **** = p < 0.0001.
(TIF)

S4 Fig Diclofenac reduces migration in HTZ-349, A172, and U87MG. Corresponding to Fig 4B, a migration decrease after diclofenac treatment was measured in all three glioma lines. Similar to ibuprofen, diclofenac treatment resulted in migration decrease in a time- and concentration-dependent manner. Regulation was significant from 24 h after treatment onset (compared to DMSO Ctrl (95% CI, **** = p < 0.0001) in HTZ-349 and A172, whereas U87MG showed resistance until 30 h. (A) Bar charts corresponding to the migration curves for HTZ-349 as shown in Fig 4B. (B) A172 responded to diclofenac to less extent. (C) In contrast to ibuprofen, U87MG cells showed resistance to all diclofenac concentrations until 30 h of exposure, when the highest concentrations (0.1 and 0.2 mM) achieved significance (*). Statistics: * = 0.05 > p ≤ 0.01, ** = 0.01 > p ≤ 0.001, *** = 0.001 > p ≤ 0.0001, **** = p < 0.0001.
(TIF)

S5 Fig Western blot quantification to Fig 6A (HTZ-349). For quantification purposes, we evaluated the Western blot from Fig 6A and two additional blots. (A) Expression of c-myc was significantly increased in a concentration-dependent manner after ibuprofen treatment. Additionally, a trend towards reduced pSTAT-3 expression was observed. (B) Likewise, distinct effects were obtained with diclofenac, as pSTAT-3 was reduced in a concentration-dependent way. In contrast to ibuprofen, diclofenac reduced c-myc expression significantly (0.2 mM), and LDH-A had a tendency towards decreased expression. Statistics: 90% CI, * = 0.1 > p ≤ 0.01, ** = 0.01 > p ≤ 0.001, **** = p < 0.0001.
(TIF)

S6 Fig Ibuprofen and diclofenac have different effects on STAT-3 signalling in A172. Protein expression in A172 cells was analyzed after incubation with increasing ibuprofen (0.5, 1, 2 mM) or diclofenac concentrations (0.05, 0.1, 0.2 mM) for 24 h. (A, B) Depending on concentration, ibuprofen and diclofenac reduced STAT-3 phosphorylation significantly without affecting total STAT-3 levels. C-myc was significantly down regulated by diclofenac, whereas ibuprofen had a tendency to increase protein expression. LDH-A was reduced, but not to significant extent. Statistics: * = 0.05 > p ≤ 0.01, ** = 0.01 > p ≤ 0.001, *** = 0.001 > p ≤ 0.0001, **** = p < 0.0001. (C) Corresponding quantitative RT-PCR revealed a significant LDH-A transcript decrease only with ibuprofen (2 mM, compared to DMSO Ctrl, 95% CI, * = 0.05 > p ≤ 0.01). A trend towards LDH-A expression decrease was observed with diclofenac (0.2 mM), whereas ASA (0.2 mM) had no effect.
(TIF)

S7 Fig Ibuprofen and diclofenac have different effects on STAT-3 signalling in U87MG. Protein expression in U87MG cells was analyzed after incubation with increasing ibuprofen (0.5, 1, 2 mM) or diclofenac concentrations (0.05, 0.1, 0.2 mM) for 24 h. (A, B) Depending on concentration, ibuprofen and diclofenac reduced STAT-3 phosphorylation significantly without affecting total STAT-3 levels. Expression of c-myc was not affected by diclofenac, whereas ibuprofen caused significant expression increase. A significant decrease, in a concentration-dependent manner, was observed for LDH-A when exposed to diclofenac, while ibuprofen did not affect protein amounts. Statistics: 95% CI, * = 0.05 > p ≤ 0.01, ** = 0.01 > p ≤ 0.001, *** = 0.001 > p ≤ 0.0001, **** = p < 0.0001. (C) Corresponding quantitative RT-PCR revealed a significant LDH-A transcript decrease only with diclofenac (0.2 mM, compared to DMSO Ctrl, 95% CI, * = 0.05 > p ≤ 0.01), while ibuprofen and ASA (0.2 mM) had no effect.
(TIF)

S8 Fig Western blot quantification corresponding to Fig 7A (HTZ-349) and mRNA expression of LDH-A after exposure to STATTIC. (A) For quantification purposes, two more blots were evaluated in addition to the exemplary Western blot from Fig 7A. In line with a decrease of STAT-3 phosphorylation at Y705, expression of c-myc was significantly reduced in a concentration-dependent manner after STATTIC treatment. Total STAT-3 expression was not altered. STATTIC proved to be specific as phosphorylation at S727 of STAT-3 was not significantly affected. Statistics: 95% CI, * = 0.05 > p ≤ 0.01, ** = 0.01 > p ≤ 0.001, **** = p < 0.0001. LDH-A protein expression was not decreased within 24 h of exposure to STATTIC. (B) However, on mRNA levels, LDH-A was significantly decreased compared to DMSO control. (C) Percentage decrease of migration was reduced at every time point. A small decrease was observed after 6 h and a strong reduction at 24 and 30 h compared to DMSO Ctrl. Statistics: 95% CI, * = 0.05 > p ≤ 0.01, *** = 0.001 > p ≤ 0.0001.
(TIF)

S9 Fig Inhibition of STAT-3 phosphorylation changes signalling patterns along with functional effects in A172. (A) Phosphorylated STAT-3 (Y705) and c-myc were decreased specifically and concentration-dependent in A172 exposed to STATTIC (compared to DMSO Ctrl, 95% CI, 10 μM: *** = 0.001 > p ≤ 0.0001, 20 μM: **** = p < 0.0001). Concurrently, total STAT-3 and STAT-3 phosphorylated at S727 remained at the same level. Additionally, LDH-A remained unchanged as well. (B) All STATTIC concentrations indicated a significant decrease of cell migration beginning at 24 h after treatment (95% CI, **** = p < 0.0001). (C) Accordingly, proliferation was decreased, with a significant decline at 20 μM (95% CI, **** = p < 0.0001).
(TIF)

S10 Fig Inhibition of STAT-3 phosphorylation altered signalling patterns along with functional effects in U87MG. (A) In U87MG exposed to STATTIC, phosphorylated STAT-3 (Y705) was decreased specifically in a concentration-dependent manner (compared to DMSO Ctrl, 95% CI, 5 μM: * = 0.05 > p ≤ 0.01, 10 μM: *** = 0.001 > p ≤ 0.0001, 20 μM: **** = p < 0.0001). Corresponding effects were observed for LDH-A (20 μM: * = 0.05 > p ≤ 0.01). Total STAT-3 and STAT-3 phosphorylated at S727 remained at the same level. Additionally, c-myc was decreased with 20 μM STATTIC (* = 0.05 > p ≤ 0.01). (B) 24 h after treatment, all STATTIC concentrations achieved a significant restriction of cell migration (95% CI, **** = p < 0.0001; 6 h: 15 μM = *** = 0.001 > p ≤ 0.0001). (C) Accordingly, proliferation was decreased, with a significant decline at 96 h with 10 and 20 μM (95% CI, **** = p < 0.0001). 48 h after treatment, 20 μM of STATTIC achieved a significant decrease (* = 0.05 > p ≤ 0.01).
(TIF)

S1 File Supplementary Materials and Methods. Actin staining and G-/F-actin measurement.
(DOCX)
