# Supplementary figures and images for: Ibuprofen and Diclofenac Restrict Migration and Proliferation of Human Glioma Cells by Distinct Molecular Mechanisms
Source: PLoS One. 2015 Oct 20;10(10):e0140613. doi: 10.1371/journal.pone.0140613 (PMC4617646; doi:10.1371/journal.pone.0140613)

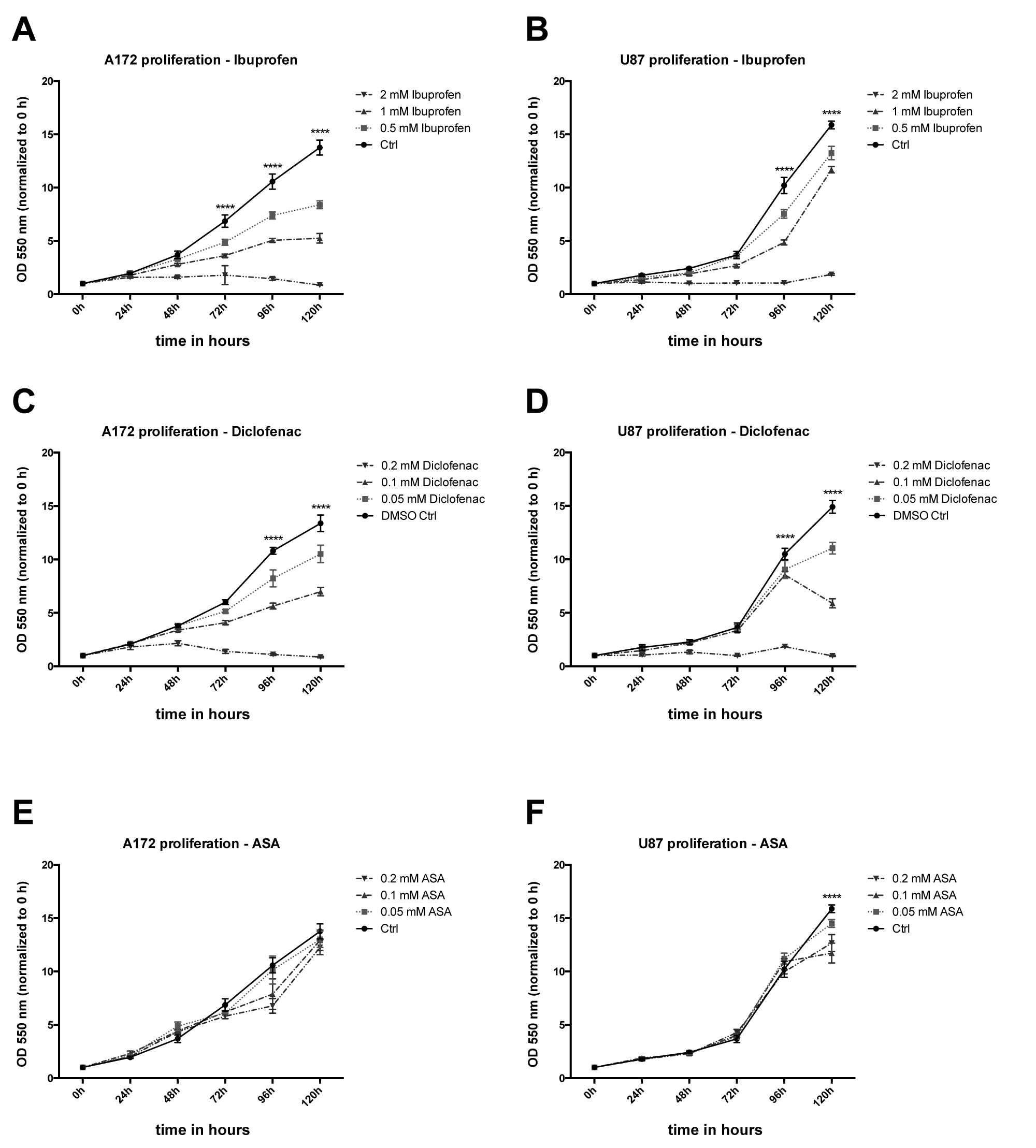

Supplement: S1 Fig — Corresponding to Fig 2, the proliferation abilities of glioma cell lines A172 and U87MG were analyzed after ibuprofen (0.5, 1, 2 mM), diclofenac (0.05, 0.1, 0.2 mM), or ASA (0.05, 0.1, 0.2 mM) treatment (A, B). At 96 and 120 h, all ibuprofen concentrations achieved significant reduction of A172’s and U87MG’s proliferation (compared to non-treated Ctrl, 95% CI, p < 0.0001). Ibuprofen proved to be more effective in A172 (A). Significant values were as follows: (A) 48 h: 2 mM = 0.001 > p ≤ 0.0001, 48 h: 1 mM = 0.05 > p ≤ 0.01; (B) 48 and 72 h: 2 mM = p < 0.0001, 72 h: 1 mM = 0.001 > p ≤ 0.0001. (C, D) Similar proliferation inhibiting effects were obtained with diclofenac. At 96 and 120 h, all concentrations resulted in a significant reduction (compared to DMSO Ctrl, 95% CI, p < 0.0001). However, diclofenac was not as effective as ibuprofen on A172 cells (compare Figs A and C). Significant values were: (C) 48 and 72 h: 2 mM = p < 0.0001, 72 h: 1 mM = p < 0.0001; (D) 48 h: 0.2 mM = 0.01 > p ≤ 0.001, 72 h: 2 mM = p < 0.0001. (E) ASA had time-dependent effects in A172 with the highest concentration of 0.2 mM (72, 96 and 120 h: 0.2 mM = 0.001 > p ≤ 0.0001), but was not as effective as diclofenac or ibuprofen. (F) ASA has neither concentration- nor time-dependent effects on U87MG cell proliferation as all ASA concentrations significantly decrease proliferation only at 120 h (significant value: 96 h: 2 mM = 0.01 > p ≤ 0.001). (TIF) [file pone.0140613.s001.tif]

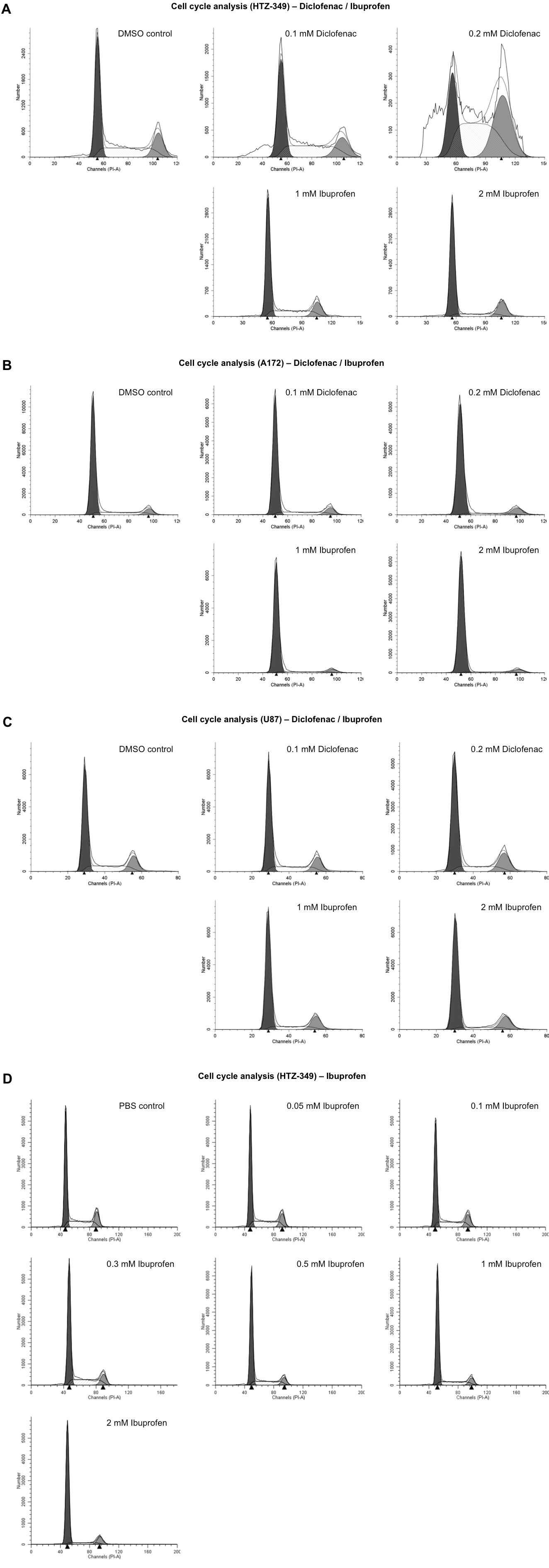

Supplement: S2 Fig — Ibuprofen and diclofenac induced cell cycle arrest in all cell lines, although at different checkpoints. The most prominent effects were observed from diclofenac treatment in HTZ-349, where increasing concentrations resulted in a sub-G1 peak, indicating cell death (Figs A and D). This was not observed in A172 (Fig B) or U87MG (Fig C). Figures depict representative histograms of each treatment. (TIF) [file pone.0140613.s002.tif]

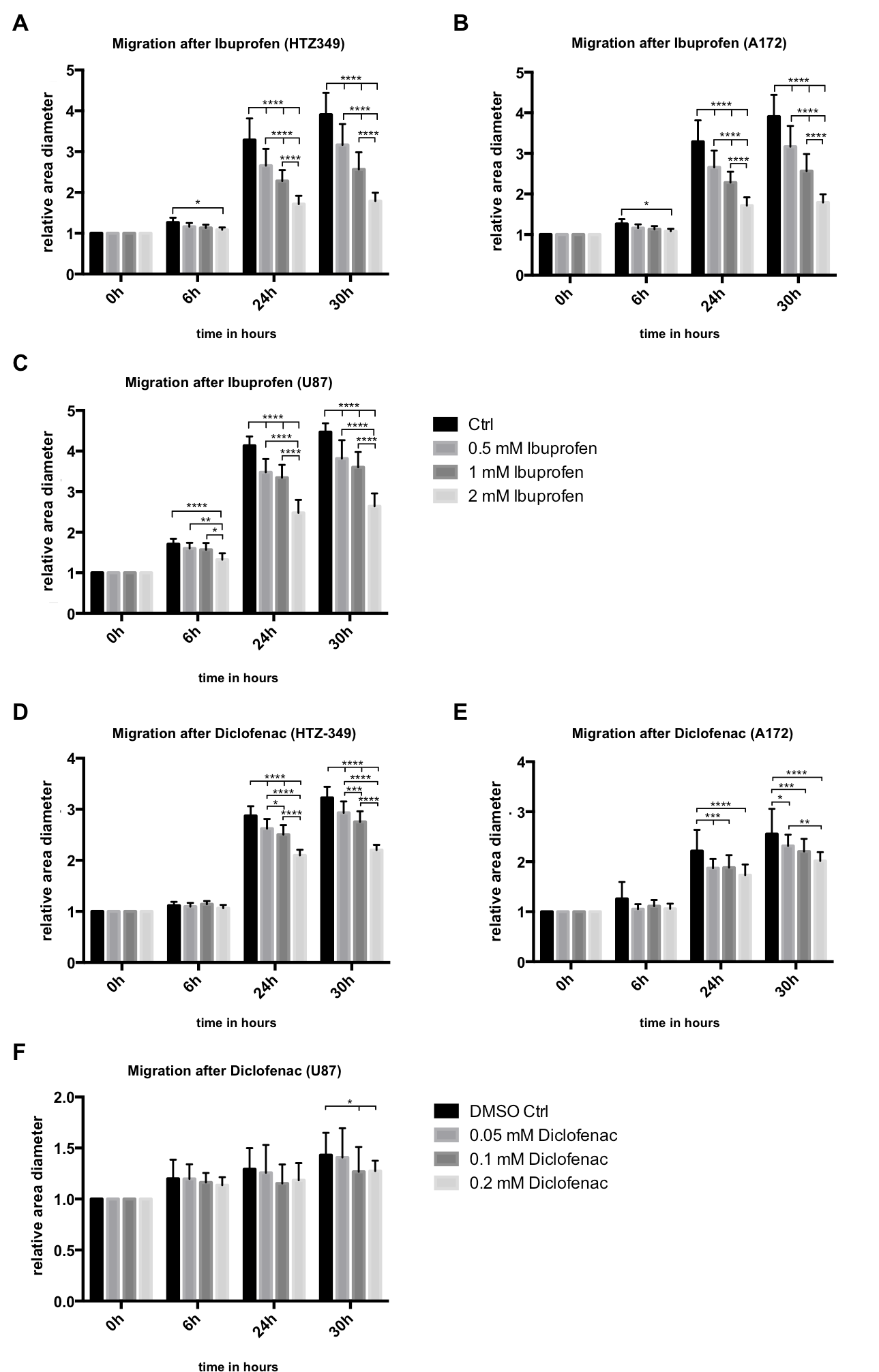

Supplement: S3 Fig — Ibuprofen decreased migration in a time- and concentration-dependent manner in all glioma lines starting 6 h after treatment compared to a non-treated control (95% CI, **** = p < 0.0001). (A) Bar charts corresponding to the migration curves for HTZ-349 as shown in Fig 4A. (B) Similar response to ibuprofen was observed for the glioma line A172. (C) Response was increased in U87MG cells as all concentrations achieved significant inhibition of migration after only 6 h of exposure to ibuprofen. Statistics: * = 0.05 > p ≤ 0.01, ** = 0.01 > p ≤ 0.001, *** = 0.001 > p ≤ 0.0001, **** = p < 0.0001. (TIF) [file pone.0140613.s003.tif]

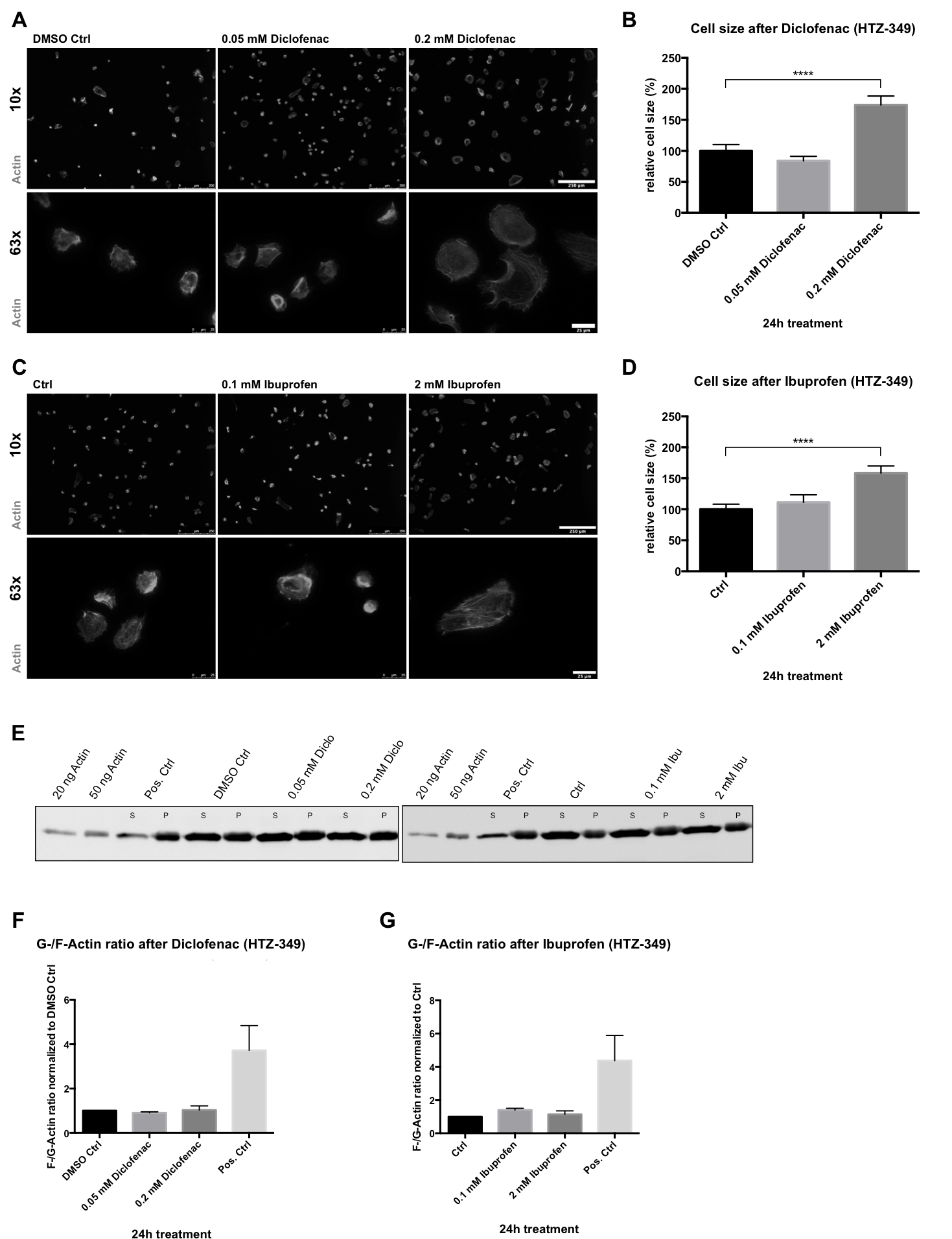

Supplement: S4 Fig — Corresponding to Fig 4B, a migration decrease after diclofenac treatment was measured in all three glioma lines. Similar to ibuprofen, diclofenac treatment resulted in migration decrease in a time- and concentration-dependent manner. Regulation was significant from 24 h after treatment onset (compared to DMSO Ctrl (95% CI, **** = p < 0.0001) in HTZ-349 and A172, whereas U87MG showed resistance until 30 h. (A) Bar charts corresponding to the migration curves for HTZ-349 as shown in Fig 4B. (B) A172 responded to diclofenac to less extent. (C) In contrast to ibuprofen, U87MG cells showed resistance to all diclofenac concentrations until 30 h of exposure, when the highest concentrations (0.1 and 0.2 mM) achieved significance (*). Statistics: * = 0.05 > p ≤ 0.01, ** = 0.01 > p ≤ 0.001, *** = 0.001 > p ≤ 0.0001, **** = p < 0.0001. (TIF) [file pone.0140613.s004.tif]

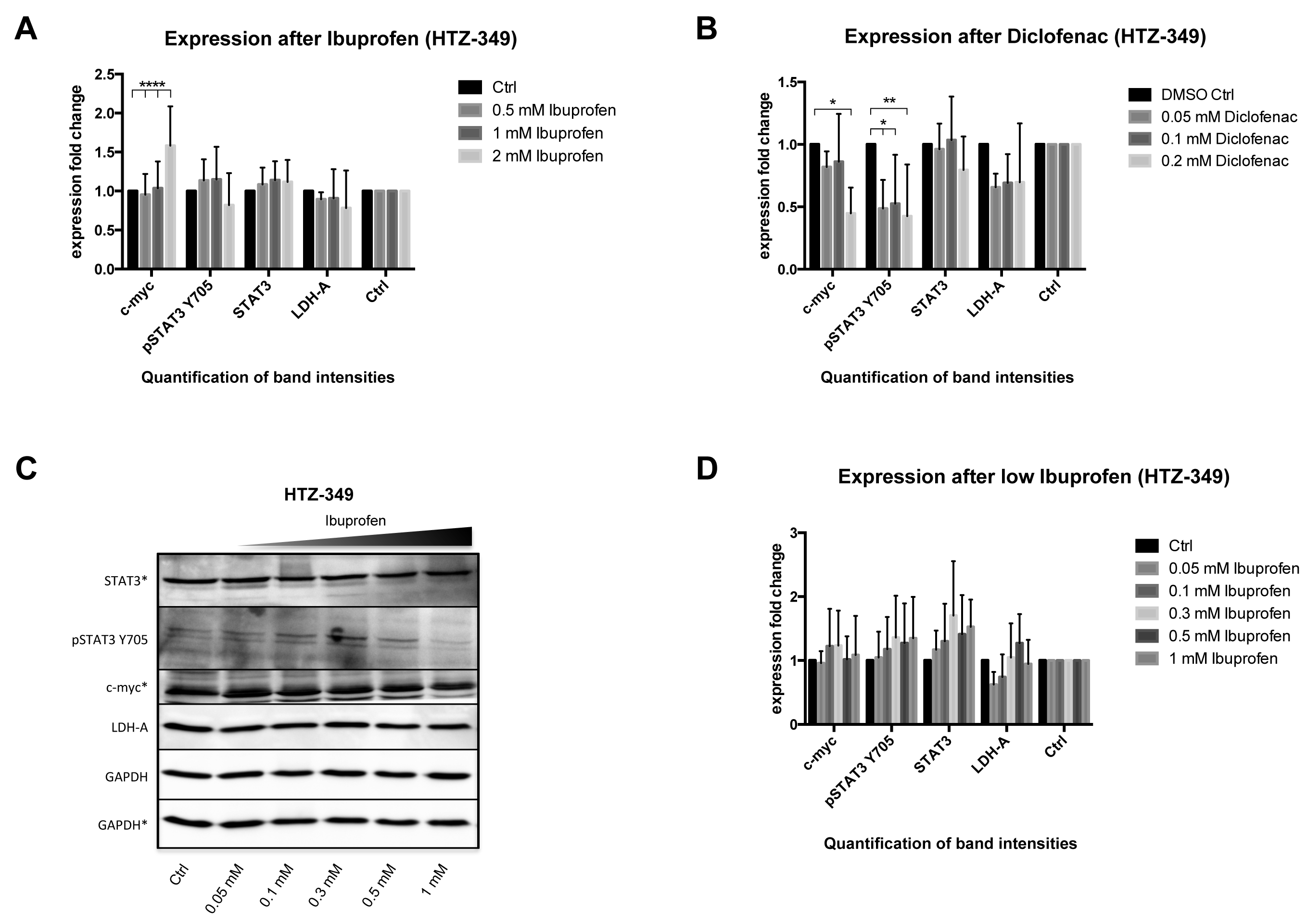

Supplement: S5 Fig — For quantification purposes, we evaluated the Western blot from Fig 6A and two additional blots. (A) Expression of c-myc was significantly increased in a concentration-dependent manner after ibuprofen treatment. Additionally, a trend towards reduced pSTAT-3 expression was observed. (B) Likewise, distinct effects were obtained with diclofenac, as pSTAT-3 was reduced in a concentration-dependent way. In contrast to ibuprofen, diclofenac reduced c-myc expression significantly (0.2 mM), and LDH-A had a tendency towards decreased expression. Statistics: 90% CI, * = 0.1 > p ≤ 0.01, ** = 0.01 > p ≤ 0.001, **** = p < 0.0001. (TIF) [file pone.0140613.s005.tif]

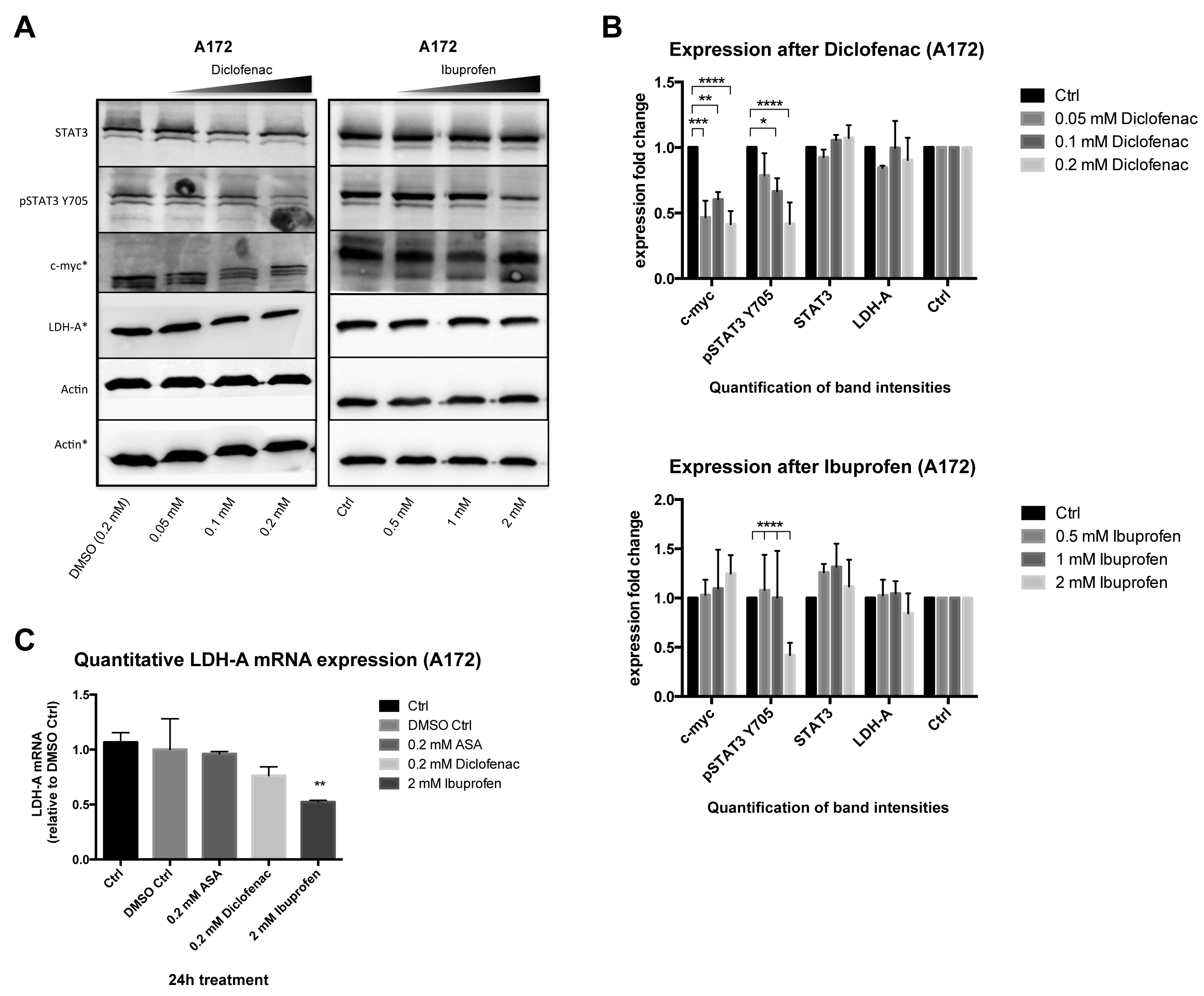

Supplement: S6 Fig — Protein expression in A172 cells was analyzed after incubation with increasing ibuprofen (0.5, 1, 2 mM) or diclofenac concentrations (0.05, 0.1, 0.2 mM) for 24 h. (A, B) Depending on concentration, ibuprofen and diclofenac reduced STAT-3 phosphorylation significantly without affecting total STAT-3 levels. C-myc was significantly down regulated by diclofenac, whereas ibuprofen had a tendency to increase protein expression. LDH-A was reduced, but not to significant extent. Statistics: * = 0.05 > p ≤ 0.01, ** = 0.01 > p ≤ 0.001, *** = 0.001 > p ≤ 0.0001, **** = p < 0.0001. (C) Corresponding quantitative RT-PCR revealed a significant LDH-A transcript decrease only with ibuprofen (2 mM, compared to DMSO Ctrl, 95% CI, * = 0.05 > p ≤ 0.01). A trend towards LDH-A expression decrease was observed with diclofenac (0.2 mM), whereas ASA (0.2 mM) had no effect. (TIF) [file pone.0140613.s006.tif]

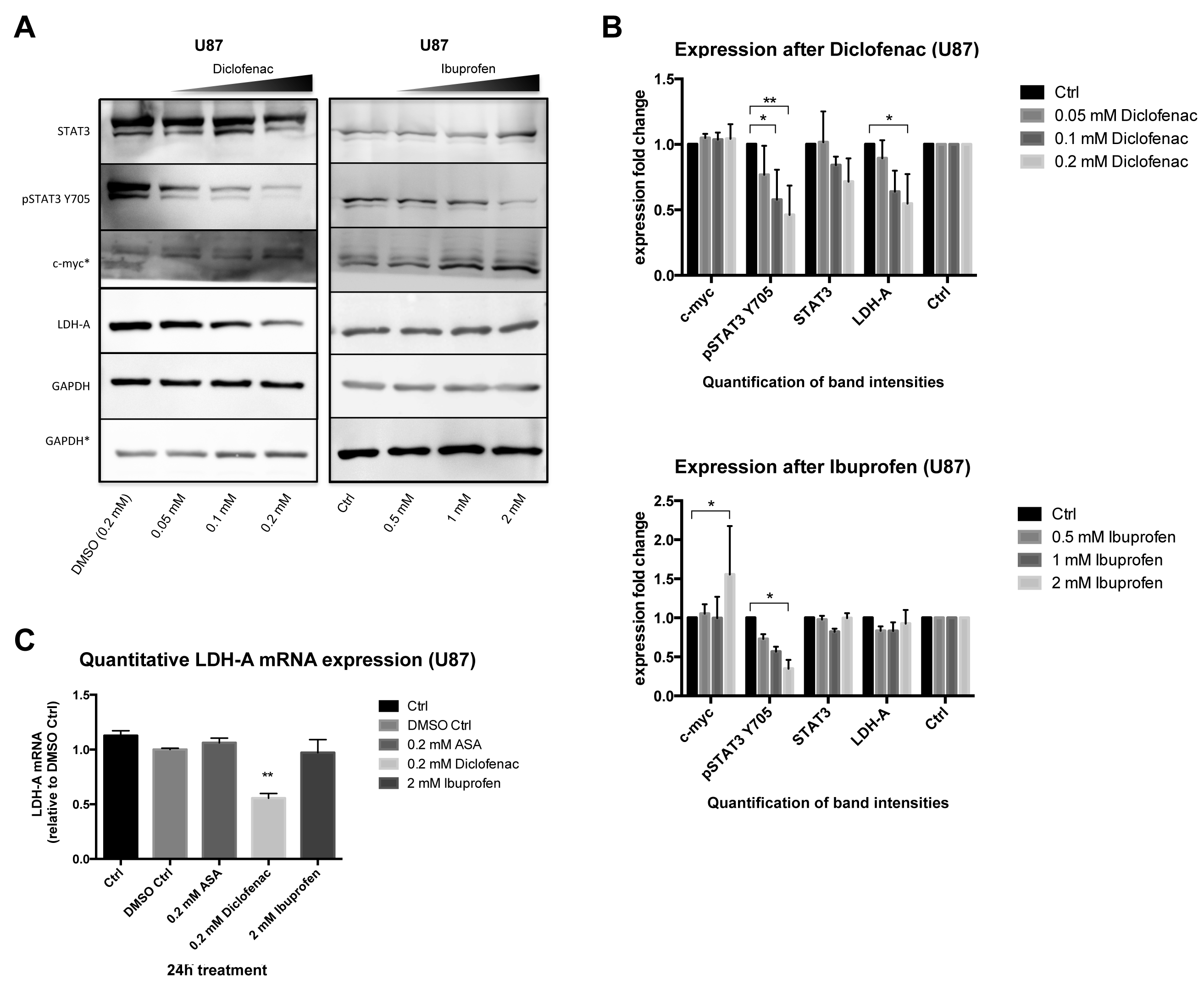

Supplement: S7 Fig — Protein expression in U87MG cells was analyzed after incubation with increasing ibuprofen (0.5, 1, 2 mM) or diclofenac concentrations (0.05, 0.1, 0.2 mM) for 24 h. (A, B) Depending on concentration, ibuprofen and diclofenac reduced STAT-3 phosphorylation significantly without affecting total STAT-3 levels. Expression of c-myc was not affected by diclofenac, whereas ibuprofen caused significant expression increase. A significant decrease, in a concentration-dependent manner, was observed for LDH-A when exposed to diclofenac, while ibuprofen did not affect protein amounts. Statistics: 95% CI, * = 0.05 > p ≤ 0.01, ** = 0.01 > p ≤ 0.001, *** = 0.001 > p ≤ 0.0001, **** = p < 0.0001. (C) Corresponding quantitative RT-PCR revealed a significant LDH-A transcript decrease only with diclofenac (0.2 mM, compared to DMSO Ctrl, 95% CI, * = 0.05 > p ≤ 0.01), while ibuprofen and ASA (0.2 mM) had no effect. (TIF) [file pone.0140613.s007.tif]

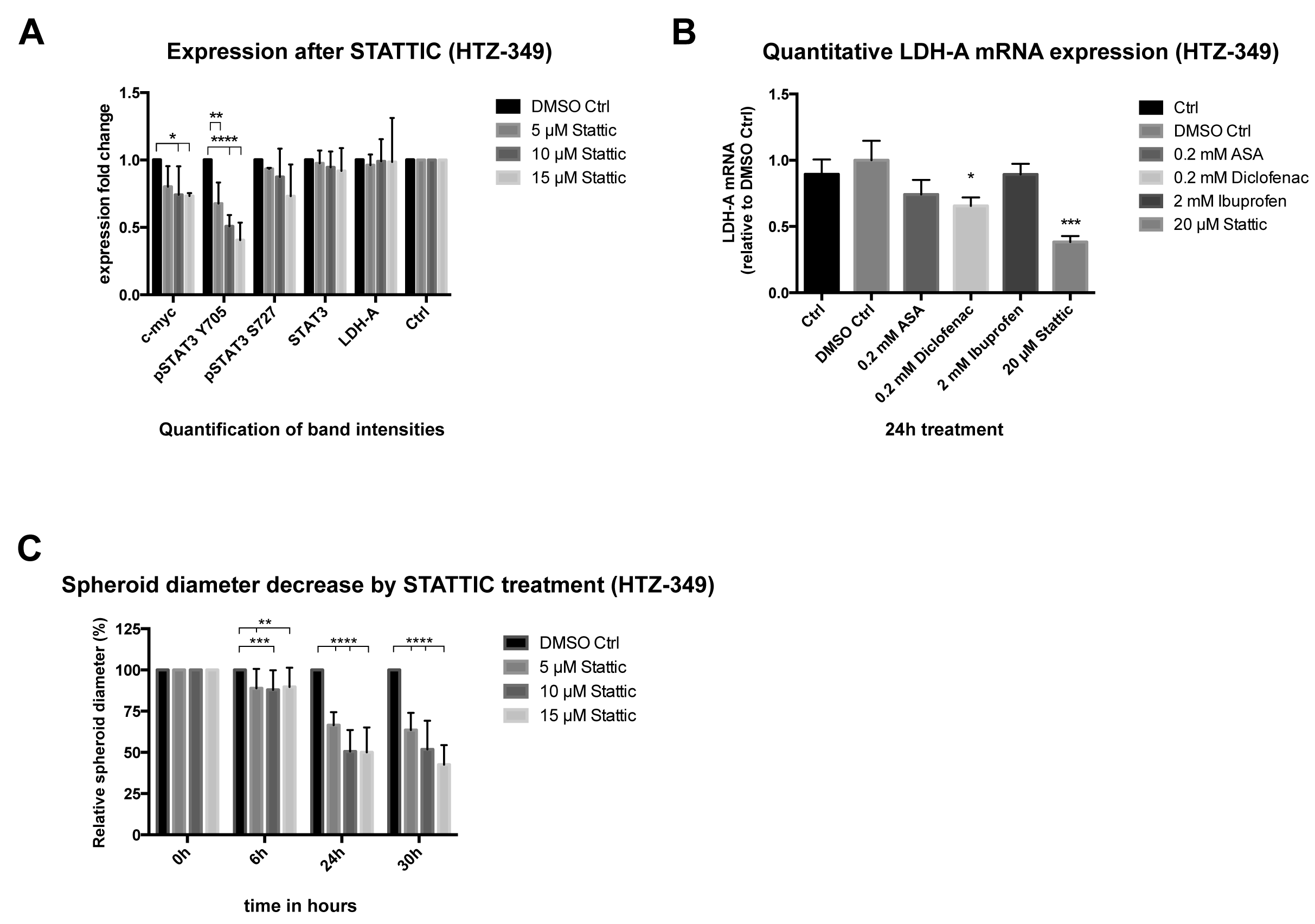

Supplement: S8 Fig — (A) For quantification purposes, two more blots were evaluated in addition to the exemplary Western blot from Fig 7A. In line with a decrease of STAT-3 phosphorylation at Y705, expression of c-myc was significantly reduced in a concentration-dependent manner after STATTIC treatment. Total STAT-3 expression was not altered. STATTIC proved to be specific as phosphorylation at S727 of STAT-3 was not significantly affected. Statistics: 95% CI, * = 0.05 > p ≤ 0.01, ** = 0.01 > p ≤ 0.001, **** = p < 0.0001. LDH-A protein expression was not decreased within 24 h of exposure to STATTIC. (B) However, on mRNA levels, LDH-A was significantly decreased compared to DMSO control. (C) Percentage decrease of migration was reduced at every time point. A small decrease was observed after 6 h and a strong reduction at 24 and 30 h compared to DMSO Ctrl. Statistics: 95% CI, * = 0.05 > p ≤ 0.01, *** = 0.001 > p ≤ 0.0001. (TIF) [file pone.0140613.s008.tif]

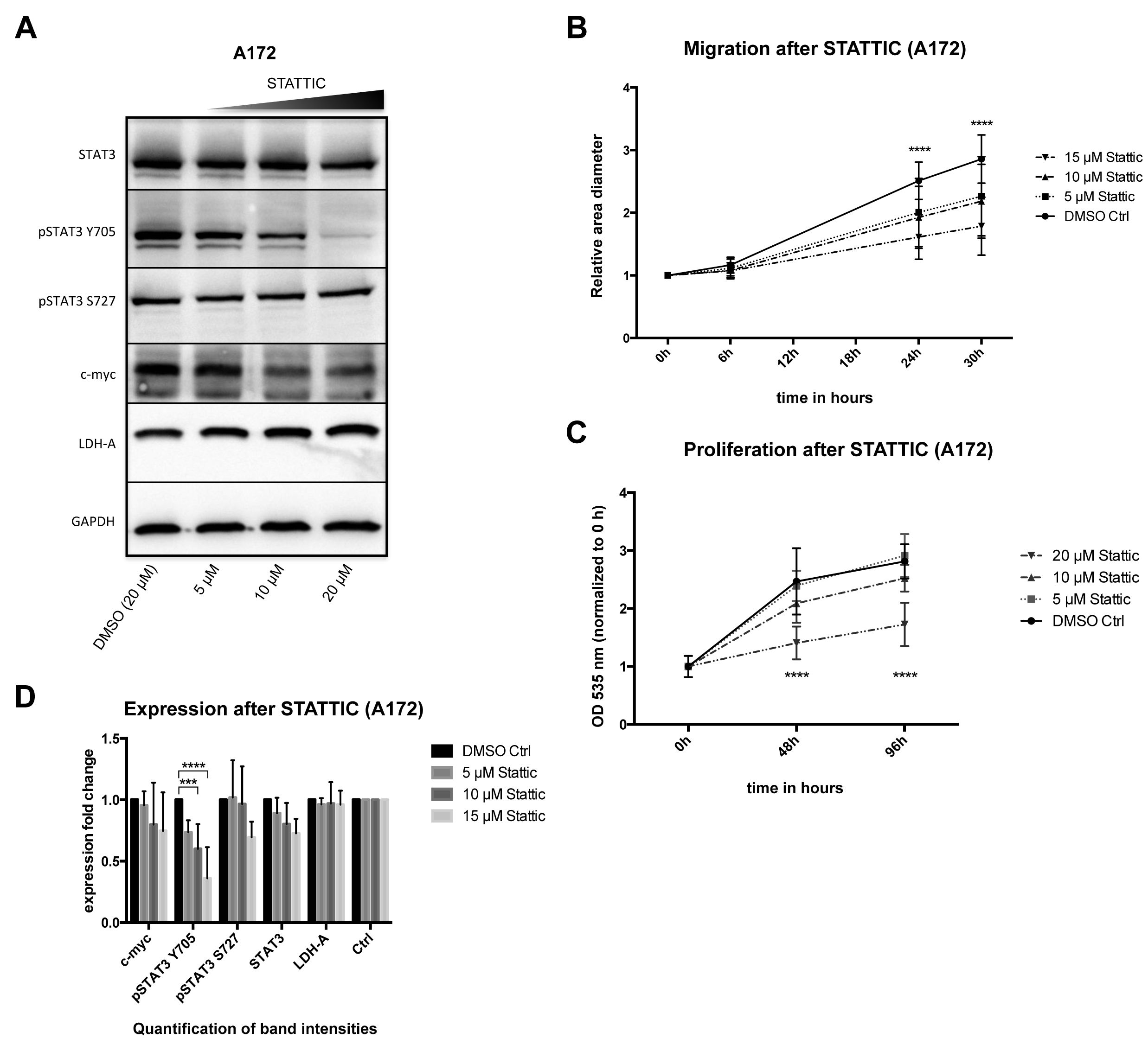

Supplement: S9 Fig — (A) Phosphorylated STAT-3 (Y705) and c-myc were decreased specifically and concentration-dependent in A172 exposed to STATTIC (compared to DMSO Ctrl, 95% CI, 10 μM: *** = 0.001 > p ≤ 0.0001, 20 μM: **** = p < 0.0001). Concurrently, total STAT-3 and STAT-3 phosphorylated at S727 remained at the same level. Additionally, LDH-A remained unchanged as well. (B) All STATTIC concentrations indicated a significant decrease of cell migration beginning at 24 h after treatment (95% CI, **** = p < 0.0001). (C) Accordingly, proliferation was decreased, with a significant decline at 20 μM (95% CI, **** = p < 0.0001). (TIF) [file pone.0140613.s009.tif]

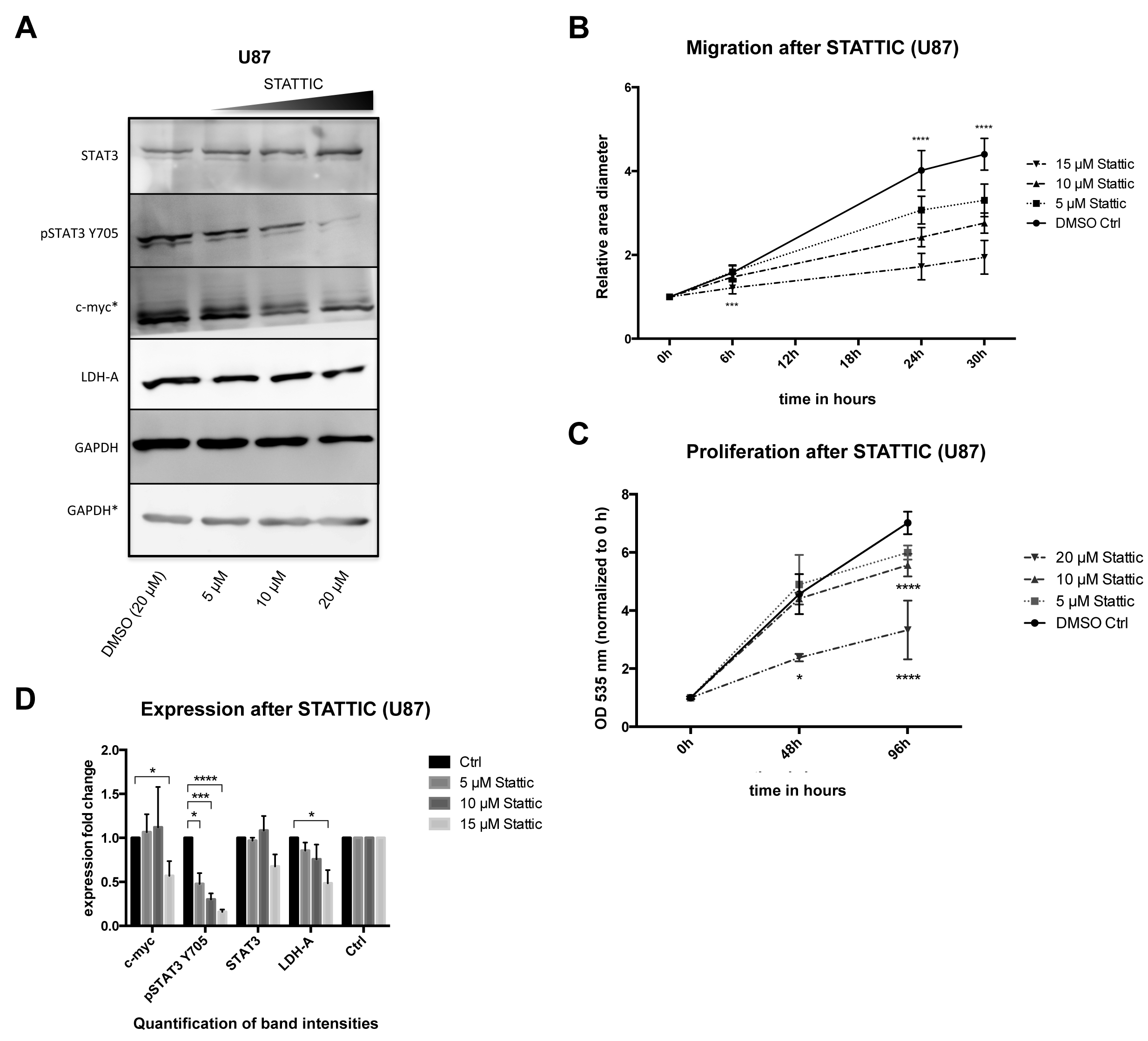

Supplement: S10 Fig — (A) In U87MG exposed to STATTIC, phosphorylated STAT-3 (Y705) was decreased specifically in a concentration-dependent manner (compared to DMSO Ctrl, 95% CI, 5 μM: * = 0.05 > p ≤ 0.01, 10 μM: *** = 0.001 > p ≤ 0.0001, 20 μM: **** = p < 0.0001). Corresponding effects were observed for LDH-A (20 μM: * = 0.05 > p ≤ 0.01). Total STAT-3 and STAT-3 phosphorylated at S727 remained at the same level. Additionally, c-myc was decreased with 20 μM STATTIC (* = 0.05 > p ≤ 0.01). (B) 24 h after treatment, all STATTIC concentrations achieved a significant restriction of cell migration (95% CI, **** = p < 0.0001; 6 h: 15 μM = *** = 0.001 > p ≤ 0.0001). (C) Accordingly, proliferation was decreased, with a significant decline at 96 h with 10 and 20 μM (95% CI, **** = p < 0.0001). 48 h after treatment, 20 μM of STATTIC achieved a significant decrease (* = 0.05 > p ≤ 0.01). (TIF) [file pone.0140613.s010.tif]
