# Supplementary material for: Ibuprofen and Diclofenac Restrict Migration and Proliferation of Human Glioma Cells by Distinct Molecular Mechanisms
Source: PLoS One. 2015 Oct 20;10(10):e0140613. doi: 10.1371/journal.pone.0140613 (PMC4617646; doi:10.1371/journal.pone.0140613)
Supplement: S1 File — Actin staining and G-/F-actin measurement. (DOCX) [file pone.0140613.s011.docx]

**Supplementary Materials and Methods**

**Actin staining**

2 x 10^4^ HTZ-349 cells were seeded on laminin (Corning, USA) coated cover slips (Thermo Scientific, USA) one day prior to treatment with diclofenac (0.05, 0.2 mM) or ibuprofen (0.1, 2 mM). After 24 h, cells were fixed with paraformaldehyde (3.7% in 1x PBS) for 20 min at 4 °C and subsequently permeabilized with 0.2% Triton X-100 (in 1x PBS) for 3.5 min at room temperature. Filamentous actin was stained with TRITC-conjugated phalloidin (Rhodamine Phalloidin, 10 U/ml, Invitrogen) for 10 min at room temperature. Afterwards, cells were mounted on microscope slides with Mowiol and analyzed at a Leica AF6000LX fluorescence microscope, equipped with Leica HCX PL FLUOTAR 10x/0.30 and Leica HCX PL APO 63x/1.3 GLYC objectives and a Leica DFC350 FX digital camera (1392 x 1040 pixels, 6.45 µm x 6.45 µm pixel size).

**G-/F-actin measurement**

1.5 x 10^5^ HTZ-349 cells were seeded in 6-well plates and incubated for 24 h prior to treatment with diclofenac (0.05, 0.2 mM) or ibuprofen (0.1, 2 mM) and corresponding controls. After 24 h cells were harvested and processed according to the G-actin/F-actin In Vivo Assay Biochem Kit (Cytoskeleton, USA) instructions. Briefly, cells were lysed and F-actin pelleted by ultracentrifugation at 100,000 x g at 37 °C for 1 h in a Optima MAX-XP ultracentrifuge (Beckman Coulter, USA) equipped with a TLA-100 rotor (fixed angle, 30°, Beckman Coulter, USA). Actin content on Western blot images was determined by ImageQuant TL Software (TL 8.1, GE Healthcare, USA) in nanograms to calculate F-/G-actin ratios.
